# Cocoa-Based Plant Matrices in Glucose Metabolism: Bioactive Compounds and Redox Signaling

**DOI:** 10.3390/antiox15060732

**Published:** 2026-06-09

**Authors:** Jose Francisco Tornero-Aguilera, Miguel López-Moreno, Carlota Valeria Villanueva-Tobaldo, Alexandra Martín-Rodríguez, Agustín Curiel-Regueros, Vicente Javier Clemente-Suárez

**Affiliations:** 1 Department of Sport Sciences, Faculty of Sport and Health Sciences, Fit Generation Research Institute, AD500 Andorra la Vella, Andorra; 2 Diet, Planetary Health and Performance, Faculty of Health Sciences, Universidad Francisco de Vitoria, 28223 Madrid, Spain; m.lopez.moreno@facultyue.es; 3 Department of Nutrition and Dietetics, Faculty of Sport and Health Sciences, Fit Generation Research Institute, AD500 Andorra la Vella, Andorra; 4 Faculty of Health Sciences, UNIE University, 28015 Madrid, Spain; 5 Faculty of Life Sciences, Universidad de Nebrija, Campus de Ciencias de la Vida, La Berzosa, 28240 Madrid, Spain; 6 Grupo de Investigación en Cultura, Educación y Sociedad, Universidad de la Costa, Barranquilla 080002, Colombia

**Keywords:** cocoa polyphenols, flavanols, redox signaling, glycemic regulation, metabolic disorders, functional food matrices, gut microbiota, postprandial glycemia

## Abstract

Cocoa-based foods are increasingly recognized as complex plant-derived matrices with potential relevance for metabolic health, driven by interactions among multiple bioactive components. Metabolic disorders, including insulin resistance and type 2 diabetes, are characterized by disturbances in glucose homeostasis, oxidative stress, and endothelial dysfunction. This narrative review critically examines the antidiabetic potential of cocoa-based plant matrices, integrating evidence from nutritional biochemistry and metabolic physiology. We analyze the specific role of cocoa flavanols in redox-sensitive signaling pathways related to nitric oxide bioavailability and insulin signaling. Furthermore, we evaluate how complementary matrix components, such as non-glycemic sweeteners, prebiotic and viscous fibers, oleic-rich lipids, and micronutrients, modulate postprandial glycemic responses, gut microbiota activity, and overall metabolic regulation. Current evidence indicates that the metabolic effects of cocoa cannot be attributed to isolated compounds but emerge from coordinated interactions within the food matrix. Understanding these multi-component dynamics is essential for the rational design of cocoa-based functional foods aimed at improving glycemic control and supporting metabolic resilience.

## 1. Introduction

Diabetes mellitus comprises a complex and heterogeneous group of metabolic disorders characterized by chronic hyperglycemia resulting from alterations in insulin secretion, insulin action, or a combination of both processes. Persistent disruption of glucose homeostasis leads to widespread disturbances in carbohydrate, lipid, and protein metabolism, which over time promote systemic oxidative stress, chronic low-grade inflammation, and progressive impairment of tissue function [1,2,3]. These metabolic alterations play a central role in the development of both acute and long-term diabetic complications, including cardiovascular disease, nephropathy, neuropathy, and retinopathy. Among the different forms of the disease, type 2 diabetes mellitus (T2D) accounts for approximately 90–95% of all cases and is strongly associated with insulin resistance, excess adiposity, physical inactivity, and dietary patterns characterized by high consumption of energy-dense, highly processed foods [4,5].

Over the past decades, the prevalence of diabetes has increased markedly worldwide, reaching levels of major public health concern. Recent epidemiological estimates indicate that more than 589 million adults were living with diabetes globally in 2024, corresponding to roughly 11% of the adult population. Projections suggest that this figure could exceed 850 million individuals by 2050 if current trajectories continue [6,7]. The impact of diabetes extends well beyond its growing prevalence. The disease imposes a substantial economic burden on healthcare systems, reduces quality of life, and is associated with a marked increase in mortality risk. Complications related to diabetes remain among the leading contributors to morbidity and death globally, with cardiovascular disease representing the primary cause of mortality in affected individuals [8]. These trends underscore the need for preventive and therapeutic strategies that address not only glycemic control but also the underlying metabolic and redox-related disturbances that drive disease progression.

Three redox concepts recur throughout the present synthesis: mitochondrial reactive oxygen species (ROS) overproduction perturbs insulin signaling in metabolically active tissues [9,10,11,12,13]; postprandial glycaemic excursions amplify systemic oxidative load and drive endothelial dysfunction independently of fasting glycemia [14,15,16]; and pancreatic β-cells are uniquely vulnerable to redox imbalance because their constitutive antioxidant buffering capacity is lower than that of comparable secretory tissues [17,18]. These concepts are developed in detail in Section 3 and are revisited in Section 4 onwards when discussing how cocoa-derived constituents intervene at each level.

Disturbances in glucose metabolism are also closely associated with postprandial glycemic excursions, reduced insulin sensitivity, and alterations in intestinal nutrient handling and hormonal signaling. Rapid digestion and absorption of refined carbohydrates can produce pronounced postprandial glucose fluctuations, generating a transient metabolic state linked to elevated oxidative stress, endothelial dysfunction, and systemic inflammatory activation [14,15]. These postprandial disturbances contribute not only to the pathophysiology of diabetes but also to the increased cardiovascular risk observed in individuals with impaired glucose metabolism. For this reason, nutritional strategies aimed at modulating postprandial glycemic responses and improving metabolic flexibility have emerged as key targets in the prevention and management of metabolic disease [16].

Within this framework, growing attention has been directed toward dietary patterns and food components capable of modulating oxidative stress and glucose metabolism through redox-related mechanisms. A substantial body of epidemiological and clinical evidence indicates that diets rich in plant-derived foods are associated with improved metabolic health and a lower risk of developing T2D and cardiovascular disease [19,20]. These protective effects are not attributable to single compounds, but rather to the combined action of multiple bioactive constituents (including polyphenols, dietary fibers, unsaturated lipids, vitamins, and minerals) that interact with metabolic and redox signaling pathways at several biological levels. Importantly, current research suggests that the physiological actions of these compounds cannot be fully understood when evaluated in isolation. Instead, their biological activity is strongly influenced by the structural and compositional characteristics of the food matrix in which they are naturally embedded, which influence their bioavailability, metabolic interactions, and downstream physiological responses [21,22].

In this context, the food matrix can be defined as the complex physical and chemical organization of a food, encompassing the interactions between nutrients and non-nutrient components that influence digestion, absorption, and metabolic responses [23]. Structural characteristics of the matrix modulate the bioavailability of bioactive compounds, regulate nutrient release during gastrointestinal digestion, and affect key metabolic pathways related to glucose homeostasis, lipid metabolism, and gut microbiota activity [21,22,24].

Among plant-derived foods, cocoa represents a particularly illustrative example of a complex nutritional matrix with potential metabolic relevance. Cocoa beans and cocoa-derived products contain a diverse array of bioactive compounds, including flavanol-type polyphenols such as epicatechin and catechin, oligomeric procyanidins, dietary fibers, methylxanthines, minerals, and lipid fractions derived from cocoa butter [25,26,27]. Importantly, the metabolic effects of cocoa cannot be attributed to individual components alone, but rather to the integrated action of these constituents within the matrix. These components interact with redox-sensitive pathways involved in endothelial function, insulin signaling, and inflammatory regulation. Cocoa flavanols have received particular attention because of their capacity to modulate nitric oxide bioavailability, improve vascular function, and affect glucose metabolism [28,29,30].

In addition to polyphenols, cocoa-based food matrices often incorporate other functional elements—including soluble fibers, alternative sweeteners, and specific lipid sources—that may further shape metabolic responses. These components can influence the kinetics of digestion, alter postprandial glycemic dynamics, and interact with gut microbiota, thereby contributing to systemic metabolic regulation. The metabolic effects associated with cocoa-containing foods therefore reflect the integrated activity of numerous constituents within the matrix rather than the isolated action of a single class of compounds [31,32].

Although interest in the metabolic properties of cocoa and related plant-based matrices has grown considerably, the mechanisms through which these complex food systems influence redox-related processes and glucose metabolism are not yet fully clarified. Questions remain regarding the relative contribution of individual bioactive compounds, the possible synergistic interactions between matrix components, and the extent to which mechanistic observations translate into clinically relevant metabolic outcomes. In addition, variability in cocoa processing methods, product formulation, and compositional characteristics complicates the interpretation of available evidence and highlights the need for more integrated analyses of cocoa-derived matrices within the broader context of metabolic health [33,34,35].

Therefore, the present narrative review aims to examine the antidiabetic potential and underlying redox-related mechanism of cocoa-based plant matrices by integrating current knowledge from nutritional biochemistry, redox biology, and metabolic research. Particular attention is devoted to the roles of cocoa polyphenols, dietary fibers, lipid fractions, micronutrients, and non-glycemic sweeteners in modulating redox balance, insulin signaling, glucose metabolism, and gut microbiota-related pathways. Emphasis is placed on the mechanistic interactions among these components within complex plant matrices and their possible implications for metabolic regulation.

By bringing together evidence from mechanistic experiments, clinical studies, and translational nutrition research, this review provides a comprehensive perspective on how cocoa-derived functional matrices may contribute to metabolic health and glycemic control. A deeper understanding of these interactions may support the development of mechanistically informed nutritional strategies and matrix-based functional food formulations aimed at preventing or attenuating metabolic disorders such as insulin resistance, prediabetes, and T2D.

## 2. Materials and Methods

This narrative review was developed through a structured literature search to identify evidence on the antidiabetic potential and underlying redox-related mechanisms of cocoa-based plant matrices and their bioactive components. Primary scientific sources were considered, including original articles, clinical trials, mechanistic studies, systematic reviews, and meta-analyses. Searches were conducted in PubMed/MEDLINE, Scopus, Web of Science Core Collection, Embase, and ScienceDirect, with complementary searches in Google Scholar when needed to identify highly cited or recent studies and to complete citation tracking.

The search strategy was structured around the main conceptual domains of the review. Keywords included “cocoa”, “cocoa flavanols”, “cocoa polyphenols”, “flavan-3-ols”, “epicatechin”, “procyanidins”, “oxidative stress”, “redox signaling”, “insulin resistance”, “glucose metabolism”, “glycemic control”, “postprandial glycemia”, “endothelial function”, “metabolic syndrome”, “type 2 diabetes”, “functional foods”, “food matrix”, “plant-based matrices”, “prebiotic fibers”, “β-glucans”, “gut microbiota”, “non-glycemic sweeteners”, “oleic acid”, and “nutritional interventions”. Controlled vocabulary terms, including MeSH terms when available, were combined with free-text terms and related synonyms to improve search sensitivity and specificity.

The primary search covered articles published from 2000 to the present, as this period encompasses most of the relevant mechanistic, clinical, and translational research on cocoa bioactives, redox biology, metabolic regulation, gut microbiota interactions, and functional food matrices in metabolic disease. Earlier seminal studies were also included when necessary to support key biochemical concepts or landmark findings. Given the mechanistic and matrix-oriented framework of this review, the search was not limited to cocoa intervention studies alone. Literature on individual components frequently present in cocoa-based matrices, such as flavanols, soluble fibers, non-glycemic sweeteners, oleic-rich lipid systems, and micronutrients, was also included when directly relevant to redox-related processes, glycemic control, insulin signaling, or metabolic regulation.

Study selection was guided by predefined criteria to ensure both relevance and methodological quality. Eligible publications were peer-reviewed articles examining cocoa bioactive compounds, cocoa-derived products, or plant-based food matrices with potential implications for metabolic health. Particular emphasis was placed on studies addressing oxidative stress, redox signaling, glucose metabolism, insulin sensitivity, endothelial function, and metabolic inflammation. Human studies, especially randomized controlled trials and observational investigations, were prioritized when available, together with systematic reviews and meta-analyses on cardiometabolic outcomes. Animal and cell-based studies were also considered when they provided mechanistic insight into pathways related to redox modulation, insulin signaling, glucose transport, mitochondrial metabolism, inflammation, or gut microbiota–host interactions. Only full-text articles published in English were included.

Studies were excluded if they did not directly address metabolic, nutritional, or redox-related aspects of cocoa or plant-derived matrices. Articles focused exclusively on agronomic, technological, sensory, or industrial processing features without clear biomedical relevance were not considered. Conference abstracts, dissertations, editorials without substantial scientific synthesis, unpublished materials, and other non-peer-reviewed documents were also excluded. Publications lacking sufficient methodological quality or meaningful mechanistic relevance to the aims of the review were likewise discarded.

The final body of literature was organized according to the thematic structure of the manuscript, including redox-related mechanisms and glycemic dysregulation in metabolic disorders, cocoa polyphenols and redox signaling, non-glycemic sweeteners and postprandial glycemic control, prebiotic and soluble fibers in metabolic regulation, oleic-rich lipid matrices, micronutrients and redox balance, multi-component synergies in cocoa-based functional matrices, and their translational relevance for antidiabetic nutritional strategies. Literature screening and selection were conducted iteratively to preserve conceptual coherence, methodological rigor, and translational relevance throughout the review.

## 3. Oxidative Stress and Glycemic Dysregulation in Metabolic Disorders

Oxidative stress represents a central pathophysiological feature of metabolic disorders characterized by impaired glucose regulation, including insulin resistance, prediabetes, and T2D [9,10]. Rather than being merely a secondary consequence of chronic hyperglycemia, oxidative stress is now recognized as an active contributor to metabolic dysfunction [9,10,12]. It participates in the progressive deterioration of insulin signaling, pancreatic β-cell integrity, vascular homeostasis, and overall metabolic flexibility [9,10]. In biological terms, oxidative stress describes a condition in which the production of ROS exceeds the buffering capacity of endogenous antioxidant systems responsible for maintaining redox balance. This imbalance may lead to oxidative damage affecting lipids, proteins, and nucleic acids, while also altering redox-sensitive intracellular signaling pathways that are tightly linked to glucose metabolism and cellular energy regulation [9,12].

Under physiological conditions, ROS should not be regarded exclusively as harmful molecules. When generated in low and tightly regulated amounts, they participate in redox signaling processes that support cellular adaptation, mitochondrial activity, and normal insulin action. The balance is disrupted in states of chronic nutrient excess, obesity, and persistent dysglycemia. In these contexts, ROS production becomes excessive and spatially dysregulated, shifting from controlled signaling toward cellular stress [9,10]. Multiple interconnected sources contribute to this increase. These include mitochondrial electron leakage during oxidative phosphorylation, activation of NADPH oxidases, enhanced formation of advanced glycation end-products, and inflammatory activation of immune cells [9,10]. The resulting metabolic environment is characterized by sustained redox imbalance, which gradually becomes mechanistically linked to the development of insulin resistance and tissue dysfunction.

### 3.1. Mitochondrial ROS Generation and Metabolic Overload

Among the major intracellular sites of oxidative stress generation in metabolic disease, mitochondria play a particularly important role. Excess availability of metabolic substrates—particularly glucose and fatty acids—increases the rate of electron delivery to the mitochondrial respiratory chain and, when electron transport capacity becomes saturated, promotes premature electron leakage at Complexes I and III. This leakage results in the monovalent reduction of molecular oxygen to superoxide (O_2_˙^−^), the primary mitochondrial ROS, rather than its complete four-electron reduction to water as occurs in normal oxidative phosphorylation [10,11]. It is important to note that it is not increased electron flow per se that elevates ROS production; rather, it is the saturation and inefficiency of electron transfer—driven by substrate overload—that redirects electrons toward superoxide formation [10,11]. In insulin-resistant conditions, this process is further exacerbated by reduced mitochondrial efficiency and diminished metabolic flexibility, which impair the ability of tissues to switch appropriately between carbohydrate and lipid oxidation. This metabolic inflexibility reinforces substrate overload and further amplifies mitochondrial ROS generation, establishing a self-perpetuating cycle of redox dysregulation and metabolic dysfunction.

The liver, skeletal muscle, and adipose tissue are particularly affected, as they represent major sites of nutrient handling and insulin-mediated metabolic regulation. In these tissues, mitochondrial ROS are not only indicators of overnutrition but also active mediators of redox-sensitive metabolic signaling [10,11]. The mitochondrial perspective is therefore critical for understanding metabolic disease. It places redox imbalance directly at the interface between nutrient overload and altered cellular physiology. Within this framework, metabolic dysfunction can be interpreted as a disturbance in cellular energy processing tightly coupled to redox dysregulation. This conceptual framework becomes particularly relevant when evaluating cocoa-based plant matrices later in the review, since any metabolic benefit should be interpreted according to its ability to influence biologically meaningful pathways rather than merely increasing overall antioxidant intake.

### 3.2. Oxidative Stress, Inflammation, and Insulin Resistance

The interaction between oxidative stress and insulin resistance is especially relevant when considering nutritional strategies aimed at improving glycemic regulation. Under normal physiological conditions, insulin binding to its receptor activates receptor tyrosine kinase activity, which initiates a downstream phosphorylation cascade through insulin receptor substrate (IRS) proteins, phosphoinositide 3-kinase (PI3K), phosphatidylinositol (3,4,5)-trisphosphate (PIP3), and protein kinase B (Akt/PKB). This signaling sequence culminates in the translocation of glucose transporter type 4 (GLUT4) to the plasma membrane of skeletal muscle and adipose tissue cells, enabling insulin-stimulated glucose uptake [11,12]. Redox imbalance disrupts this pathway at multiple levels. Elevated ROS levels activate stress-sensitive serine/threonine kinases—including c-Jun N-terminal kinase (JNK), inhibitor of κB kinase β (IKKβ), and p38 mitogen-activated protein kinase (p38 MAPK)—which promote inhibitory serine phosphorylation of IRS-1 and IRS-2 [12,13]. This modification blunts the propagation of the insulin signal through PI3K/Akt and thereby reduces GLUT4 translocation and glucose uptake in peripheral tissues [12]. It should be acknowledged that the relationship between ROS and the cellular antioxidant machinery is biphasic: under low to moderate oxidative stimuli, ROS act as redox signals that promote the Keap1–Nrf2 pathway and upregulate the expression of antioxidant enzymes such as SOD, catalase, GPx, heme oxygenase-1 (HO-1), and NAD(P)H quinone oxidoreductase 1 (NQO1), constituting an adaptive (hormetic) response [13,17]. By contrast, sustained or excessive ROS exposure, as observed in chronic metabolic disease, may overwhelm this adaptive capacity and ultimately reduce the expression or activity of these enzymes—including SOD, catalase, and GPx—further impairing the cellular capacity to buffer the oxidative load [12,13]. However, it should be noted that a reduction in a specific antioxidant enzyme does not necessarily reflect a decline in total antioxidant defense, which integrates both enzymatic and non-enzymatic components such as glutathione (GSH) and vitamins C and E [12]. At the same time, redox imbalance is closely intertwined with inflammatory signaling pathways, and this convergence further amplifies insulin resistance. Rather than acting as an isolated biochemical disturbance, redox dysregulation operates within an integrated metabolic–inflammatory network that compromises glucose disposal and systemic metabolic regulation [10,12].

This relationship becomes particularly evident in obesity-associated metabolic dysfunction. During states of sustained positive energy balance, adipose tissue expansion is accompanied by cellular stress, altered adipokine secretion, immune cell infiltration, and chronic low-grade inflammation. Pro-inflammatory mediators such as TNF-α and IL-6 have long been implicated in insulin resistance, and oxidative stress contributes to their expression through redox-sensitive transcriptional mechanisms, including NF-κB activation [12,13]. In turn, inflammatory signaling enhances ROS generation and further disrupts insulin signaling pathways. This reciprocal amplification between oxidative stress and inflammation illustrates that insulin resistance is not simply a defect in glucose transport but a systemic disturbance involving complex immune–metabolic crosstalk [12,13].

### 3.3. Pancreatic β-Cell Vulnerability and the Progression Toward Overt Diabetes

Oxidative stress also plays a decisive role in the progressive deterioration of pancreatic β-cell function. These endocrine cells exhibit a particular vulnerability to redox imbalance because they express comparatively low levels of several antioxidant enzymes, which limits their ability to neutralize excess ROS [17]. This limitation becomes especially problematic under conditions of chronic glucotoxicity and lipotoxicity. Persistent elevations in circulating glucose and fatty acids promote redox imbalance within pancreatic islets, disrupt mitochondrial ATP production, impair glucose-stimulated insulin secretion, and promote β-cell dysfunction [17,18]. Over time, these disturbances contribute to the reduction in β-cell functional mass that characterizes the transition from compensated insulin resistance to overt T2D. In this context, redox dysregulation provides a mechanistic link between peripheral insulin resistance and endocrine pancreatic failure, positioning it at the center of disease progression [17,18].

This β-cell perspective also deepens the understanding of metabolic disease pathogenesis. Peripheral insulin resistance alone does not fully explain the development of diabetes; sustained hyperglycemia ultimately requires progressive impairment of β-cell compensatory capacity. Oxidative stress contributes simultaneously to both aspects of this process. It impairs insulin signaling in peripheral tissues while also reducing the ability of pancreatic cells to mount an adequate compensatory response. Such dual involvement reinforces its relevance as a central mechanistic target in nutritional strategies aimed at early metabolic intervention.

### 3.4. Hyperglycemia, Glycemic Variability, and Postprandial Oxidative Stress

Hyperglycemia itself represents a powerful driver of oxidative stress, and this relationship extends beyond chronically elevated mean glucose levels. Acute glucose excursions (particularly postprandial spikes and oscillating glycemic patterns) appear to exert pronounced effects on redox homeostasis [14]. Experimental and clinical evidence indicates that intermittent exposure to high glucose concentrations may induce greater oxidative stress and endothelial dysfunction than stable hyperglycemia of comparable average magnitude [15]. These observations suggest that glycemic variability represents a dynamic and clinically relevant contributor to redox-mediated metabolic stress, complementing the effects of sustained hyperglycemia.

This concept shifts the focus of metabolic dysregulation from static fasting glycemia toward the dynamic nature of postprandial metabolism. It also highlights that the structural characteristics of foods, the kinetics of carbohydrate digestion, and the composition of dietary matrices are key determinants of metabolic responses. Dysregulated postprandial glycemia can amplify oxidative stress and contribute to cumulative damage in metabolically active tissues. Consequently, dietary matrices capable of moderating these excursions may confer benefits that extend beyond simple reductions in blood glucose levels, by attenuating postprandial oxidative stress and modulating redox-sensitive pathways involved in endothelial function, inflammation and metabolic stability [14,15,16].

This issue becomes particularly relevant within modern dietary environments characterized by frequent consumption of highly processed foods rich in refined, rapidly absorbable carbohydrates. Under such conditions, glycemic variability may represent a recurring oxidative stimulus that accumulates long before clinical diabetes becomes evident. Within this framework, cocoa-based plant matrices provide a relevant model linking metabolic pathophysiology with food design. It suggests that the metabolic relevance of a dietary matrix depends not only on its intrinsic bioactive composition but also on its capacity to influence digestion, absorption, and postprandial metabolic responses.

### 3.5. Endothelial Dysfunction and Cardiometabolic Consequences

The vascular endothelium is particularly sensitive to the combined effects of redox imbalance and metabolic dysregulation. Endothelial dysfunction is recognized as an early event in diabetes-associated vascular disease, and oxidative stress plays a central role by reducing nitric oxide bioavailability, promoting inflammatory signaling, and favoring a pro-atherogenic phenotype [10,14]. Because cardiovascular disease represents the leading cause of morbidity and mortality in T2D, the metabolic significance of oxidative stress must be interpreted not only in relation to glucose regulation but also with respect to vascular outcomes [8,10]. Within this pathophysiological context, cocoa-based matrices are of particular interest, since some of the most consistently reported biological effects of their flavanol fraction involve improvements in endothelial function, nitric oxide-dependent signaling, and vascular reactivity. These mechanisms directly target pathways implicated in cardiometabolic complications, highlighting the potential relevance of nutritional strategies aimed at modulating redox-sensitive vascular processes.

Given that oxidative stress contributes simultaneously to dysglycemia and vascular dysfunction, nutritional strategies capable of influencing both domains represent a relevant target for functional food development. Cocoa has received considerable attention in this regard because its flavanol fraction has been extensively investigated for its effects on endothelial responses, vascular tone, and nitric oxide signaling. These mechanisms are particularly important within the integrated framework of oxidative stress and glycemic dysregulation, where vascular and metabolic alterations are tightly interconnected.

### 3.6. Interpreting Redox Biology Beyond Simplistic Antioxidant Claims

The role of oxidative stress in metabolic disease requires careful mechanistic interpretation rather than simplistic assumptions regarding antioxidant effects. The presence of oxidative stress does not imply that indiscriminate antioxidant interventions will confer metabolic benefit, as redox biology is governed by tightly regulated signaling processes. Redox biology is intrinsically complex. Reactive oxygen species also serve important signaling functions, and their complete suppression may disrupt redox-sensitive pathways involved in cellular adaptation and metabolic regulation. Within this context, cocoa and other plant-derived matrices should not be interpreted simplistically as generic “antioxidant foods.” What matters biologically is not merely the radical scavenging capacity measured in chemical assays but the ability of dietary components to influence relevant physiological pathways, including redox-sensitive signaling, inflammatory responses, endothelial function, mitochondrial adaptation, and glycemic dynamics [9,10].

From both methodological and translational perspectives, this clarification is essential. Many food-derived compounds exhibit strong antioxidant activity in vitro, yet such findings do not automatically translate into measurable physiological effects in humans. The critical question for cocoa-based matrices is therefore whether their bioactive constituents can modulate redox-related mechanisms in vivo to influence metabolic health. Addressing this question requires moving beyond reductionist interpretations toward a more integrated understanding of food structure, bioavailability, and metabolic context.

### 3.7. Relevance for Nutritional Strategies and Transition Toward Cocoa-Based Matrices

From a translational standpoint, the mechanistic connections between oxidative stress and glycemic dysregulation support the development of functional food matrices targeting multiple interconnected metabolic pathways. An effective nutritional strategy should not be limited to reducing acute glucose excursions but should also aim to attenuate glycemic variability, lower oxidative burden, improve endothelial responses, and enhance overall metabolic resilience through coordinated effects on redox-sensitive pathways.

Plant-derived matrices are particularly interesting in this regard because they often integrate several classes of bioactive compounds, including polyphenols, dietary fibers, unsaturated lipids, and micronutrients, that interact with overlapping metabolic pathways [19,20,21,22,23]. Their biological effects include modulation of carbohydrate digestion and absorption, reduced postprandial insulin demand, modulation of gut-derived signaling, and regulation of redox-sensitive inflammatory networks. These characteristics are directly applicable to cocoa-based matrices, whose metabolic effects arise from the integrated activity of multiple constituents rather than from a ¡ single isolated compound.

Within this framework, cocoa represents a relevant model for studying matrix-based metabolic effects. It contains flavanol-rich polyphenols that have been widely investigated for their role in redox regulation and vascular function, and it can also be incorporated into food systems that include fibers, alternative sweeteners, and lipid components capable of modulating postprandial glycemia and insulin sensitivity [25,26,27,28,29,30]. However, the potential benefits of cocoa-based matrices must be interpreted within the broader pathophysiological context described in this section. Metabolic disease involves intertwined disturbances in redox homeostasis, insulin signaling, β-cell vulnerability, and glycemic variability. Consequently, the relevance of cocoa-based products depends not only on their phytochemical composition but also on whether they can meaningfully influence these interconnected mechanisms in humans.

**Figure 1:**
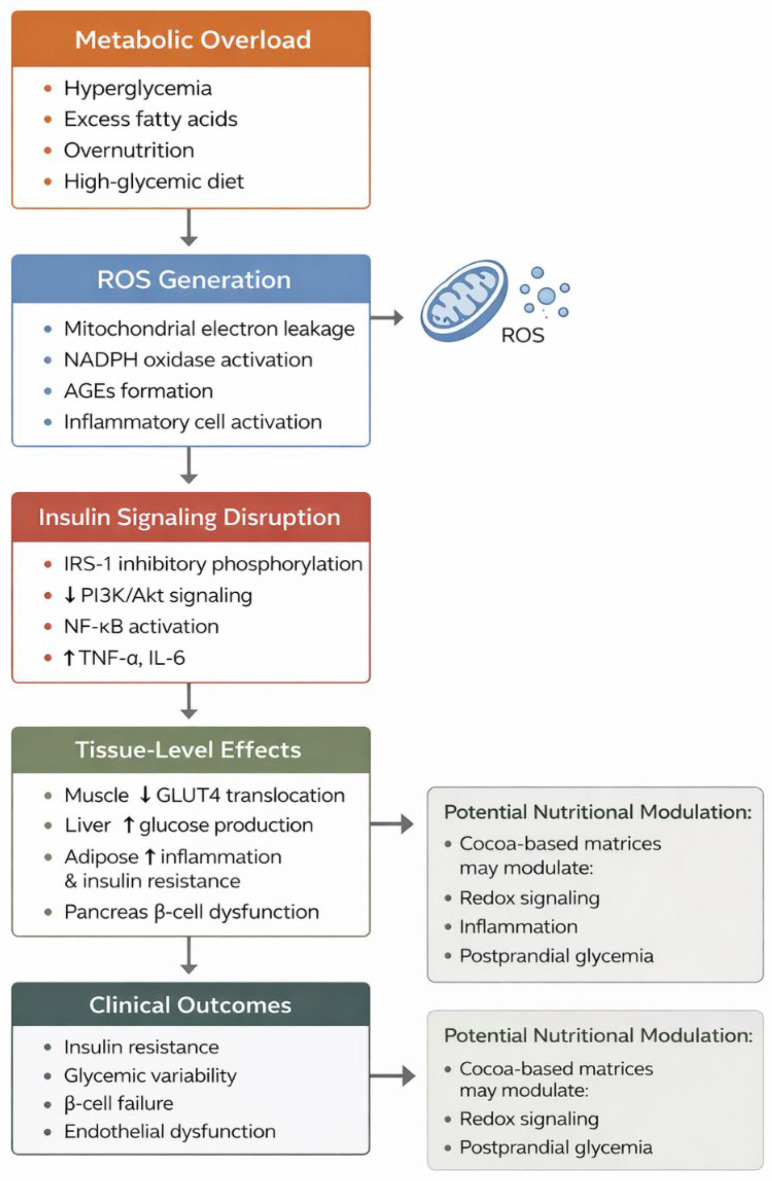
Oxidative stress as a mechanistic link between metabolic overload and glycemic dysregulation in metabolic disorders. Metabolic overload increases reactive oxygen species (ROS) production through mitochondrial dysfunction, NADPH oxidase activation, and AGE formation, leading to disruption of insulin signaling and activation of inflammatory pathways. These mechanisms contribute to insulin resistance, β-cell dysfunction, and endothelial impairment, while plant-derived matrices such as cocoa-based formulations may modulate redox signaling and postprandial metabolic responses.

Overall, oxidative stress can be understood as a mechanistic bridge linking nutrient excess with progressive metabolic dysfunction. It contributes to insulin resistance, impairs β-cell function, amplifies inflammatory signaling, and promotes endothelial dysfunction, while also being closely associated with dysregulated postprandial glycemia and glycemic variability [10,11,12,13,14,15,17,18]. This integrated perspective provides the biological framework for evaluating the metabolic relevance of cocoa-based plant matrices, as illustrated in Figure 1.

## 4. Cocoa Polyphenols and Redox Signaling in Glucose Metabolism

Cocoa is among the most concentrated dietary sources of flavan-3-ols, a characteristic that underpins its relevance as a metabolically active nutritional matrix [25,26,27,36,37,38]. The main polyphenolic compounds present in cocoa include monomeric flavanols such as (−)-epicatechin and (+)-catechin, together with oligomeric and polymeric procyanidins. Their abundance varies substantially depending on cultivar, fermentation processes, roasting conditions, alkalization, and the final formulation of cocoa products [25,27,38]. Within the scope of this review, the relevance of cocoa polyphenols does not lie in a simplistic interpretation centered on chemical antioxidant capacity. Instead, their importance resides in the ability to influence redox-sensitive signaling pathways involved in endothelial physiology, insulin signaling, postprandial metabolism, and cellular responses to nutrient overload [37,38,39,40]. This distinction is essential as in metabolic disorders, the key issue is not whether cocoa flavanols neutralize reactive species in vitro, but whether they modulate biologically relevant pathways in vivo that ultimately affect glucose homeostasis and metabolic resilience [28,37,38,41].

### 4.1. Cocoa Flavanols as Metabolically Active Phytochemicals

Evidence supporting the biological activity of cocoa flavanols spans mechanistic, preclinical, and clinical research, although the robustness of this evidence varies depending on the physiological endpoint evaluated [36,37,41,42,43,44,45,46]. Early controlled human interventions demonstrated that short-term consumption of flavanol-rich dark chocolate can improve insulin sensitivity and reduce blood pressure in healthy individuals, whereas white chocolate produced no comparable changes [36]. The relevance of this work lies not only in the metabolic outcomes observed but also in the experimental comparison between dark and white chocolate, which indirectly highlights the importance of polyphenol content rather than chocolate itself as a generic food category. A subsequent study in individuals with hypertension and impaired glucose tolerance reported that the consumption of dark chocolate improved endothelial function, lowered blood pressure, and was associated with more favorable insulin-related indices [42]. These findings reinforced the idea that cocoa flavanols may influence metabolic regulation at the intersection between vascular and glucose homeostasis.

Such early clinical trials did not demonstrate that cocoa acts as a therapeutic agent for diabetes. Rather, they suggest a plausible translational pathway through which cocoa polyphenols could indirectly influence glucose metabolism via redox-sensitive endothelial and insulin-signaling mechanisms [36,42]. Later reviews by Martín, Goya, and Ramos further emphasized that cocoa flavanols may target several key features of metabolic dysfunction, including oxidative stress, inflammation, altered insulin signaling, and impaired glucose transport, while also emphasizing the heterogeneity of intervention studies and the need for mechanistic rigor when interpreting reported effects [37,38].

### 4.2. Redox Signaling Rather than Simple Antioxidant Action

A recurring misconception in cocoa research is the tendency to equate flavanol content with nonspecific “antioxidant power”. A more mechanistically grounded interpretation recognizes that cocoa polyphenols act primarily as modulators of redox-sensitive signaling networks rather than as passive radical scavengers. These networks regulate vascular tone, inflammatory activation, cellular glucose uptake, and metabolic adaptation [37,38,47]. This perspective is consistent with current understanding of dietary polyphenols as bioactive compounds that interact with enzymes, transport systems, and cellular signaling pathways, rather than functioning solely through direct antioxidant activity measured in vitro [39,40].

One of the most studied examples of this signaling-oriented mechanism involves the interaction between cocoa flavanols and nitric oxide metabolism. Several investigations suggest that cocoa flavanols enhance nitric oxide bioavailability by stimulating endothelial nitric oxide synthase activity while simultaneously reducing oxidative inactivation of nitric oxide [41,44,47]. These effects improve endothelial function and vascular responsiveness. This mechanism has direct implications for glucose metabolism. Efficient insulin-mediated glucose disposal partly depends on adequate microvascular recruitment and tissue perfusion. Impaired endothelial signaling limits the delivery of insulin and glucose to peripheral tissues, thereby contributing to metabolic dysfunction even when alterations are primarily described in glycemic terms [43,47].

### 4.3. Endothelial Function, Nitric Oxide, and Glucose Handling

The vascular dimension of cocoa flavanol action is one of the most consistent findings in the literature. Short-term cocoa consumption in patients with essential hypertension has been shown to enhance insulin-mediated vasodilation, without a corresponding reduction in whole-body insulin resistance assessed by hyperinsulinemic clamp techniques [43]. This observation is mechanistically informative because vascular responsiveness and systemic insulin sensitivity represent related but distinct aspects of metabolic regulation. Cocoa flavanols may influence endothelial components of the glucose regulatory system before measurable changes occur in classical metabolic indices such as fasting glucose or insulin resistance [43]. This distinction may partly explain why human intervention studies often report improvements in endothelial biomarkers while glycemic endpoints remain unchanged.

Meta-analytic evidence supports this interpretation. Hooper and collaborators reported that interventions involving chocolate, cocoa, or flavan-3-ols consistently improved flow-mediated dilation and showed favorable, although more modest, effects on insulin and HOMA-IR across randomized trials [29]. Similarly, Shrime et al. documented beneficial effects of flavonoid-rich cocoa on several cardiovascular risk markers, including vascular outcomes [30]. Later quantitative syntheses, including those conducted by Lin and colleagues, confirmed that cocoa flavanols can influence cardiometabolic biomarkers, although the magnitude of the effect varies according to dose, duration of intervention, baseline metabolic phenotype, and product formulation [45].

Taken together, these findings indicate that cocoa polyphenols exert particularly consistent effects on vasculometabolic pathways. Improvements in endothelial function may subsequently influence glucose homeostasis through better microvascular perfusion and insulin delivery, especially in insulin-resistant states where vascular dysfunction and dysglycemia frequently coexist [41,43,44,45,46].

### 4.4. Intracellular Signaling Pathways and Glucose Transport

Beyond vascular effects, cocoa polyphenols have also been investigated for their ability to influence intracellular pathways associated with glucose uptake and metabolic regulation. Experimental studies by Martín and colleagues demonstrated that cocoa flavanols can improve insulin responsiveness in hepatocytes, reduce hepatic glucose production, and strengthen antioxidant defenses in pancreatic β-cells exposed to oxidative stress [37]. These findings extend the biological plausibility of cocoa beyond vascular physiology, suggesting effects on both insulin-responsive tissues and insulin-secreting cells. Additional evidence supports the involvement of cocoa flavanols in pathways regulating glucose transport and insulin signaling, including enhanced peripheral insulin signaling, and attenuation of oxidative-inflammatory processes associated with T2D [48].

Research by Yamashita and collaborators has further clarified possible molecular mechanisms. In a mouse model, cacao liquor procyanidin extract improved glucose tolerance by enhancing Glucose Transporter Type 4 (GLUT4) translocation and glucose uptake in skeletal muscle [47]. In a subsequent study, trimeric and tetrameric procyanidins activated both insulin-dependent and AMP-activated protein kinase (AMPK)-related pathways, leading to increased GLUT4 translocation and suppression of acute hyperglycemia in a dose-dependent manner [38].

These studies identify specific signaling mechanisms, moving beyond generalized antioxidant explanations. However, their translational relevance requires careful considerations, as many of these findings originate from experimental models using purified fractions or doses that do not necessarily correspond to typical human dietary exposure [48,49]. Nonetheless, they provide a mechanistic framework supporting the possibility that cocoa procyanidins can modulate skeletal muscle glucose uptake through both insulin-dependent and insulin-independent pathways.

### 4.5. Acute Metabolic Stress and Postprandial Redox Protection

An important aspect of cocoa research concerns its capacity to modulate postprandial oxidative stress, a condition closely linked to glycemic variability and acute glucose excursions. Evaluating cocoa under postprandial conditions provides insight into its effects on dynamic metabolic stress responses rather than static glycemic measures. I. In this context, Grassi et al. demonstrated that flavanol-rich dark chocolate attenuated endothelial dysfunction and oxidative stress induced by an oral glucose tolerance test, whereas white chocolate produced no comparable effect [41].

Instead of simply lowering baseline glucose levels, cocoa flavanols appeared to improve the physiological response to an acute metabolic challenge [41]. Such findings align with broader analyses, including those by Strat and colleagues, who reviewed potential mechanisms through which cocoa flavanols may influence metabolic syndrome-related disturbances. These mechanisms include interactions with digestive enzymes, glucose transporters, endothelial function, oxidative stress pathways, and inflammatory signaling [49]. The relevance of this work lies in its integrative interpretation. Cocoa flavanols may not act through a single dominant mechanism but rather through multiple modest effects that collectively influence postprandial glucose excursions and metabolic stress responses. This multi-target profile is compatible with the concept of cocoa as a functional food matrix rather than a single-compound pharmacological intervention.

### 4.6. Human Evidence: Promise, Inconsistency, and Context Dependence

Despite the mechanistic plausibility and several encouraging trials, human evidence remains heterogeneous. Dicks and colleagues reported that regular intake of a typical serving of flavanol-rich cocoa powder did not significantly improve glucose metabolism, glycated hemoglobin (HbA1c), insulin concentrations, or the Homeostatic Model Assessment of Insulin Resistance (HOMA-IR) in patients with established type 2 diabetes and hypertension receiving stable medical treatment [50]. These findings temper overly optimistic interpretations of cocoa as a universal metabolic intervention, suggesting that in advanced or pharmacologically managed disease states, the additional effects of cocoa flavanols may be limited or difficult to detect over short intervention periods [40].

The variability observed across studies reflects the influence of multiple contextual factors, including participant phenotype, medication use, habitual diet, flavanol dose, intervention duration, and product composition.

Importantly, this heterogeneity does not negate the biological relevance of cocoa polyphenols but rather defines the conditions under which their effects are more likely to be observed. Cocoa polyphenols appear to produce more consistent effects on endothelial and redox-related vascular outcomes than on traditional glycemic endpoints such as fasting glucose or HbA1c, particularly in short-term studies [40,41,43,44,45,46]. Another important source of heterogeneity arises from differences in cocoa products themselves. A flavanol-standardized cocoa beverage, a commercial chocolate product rich in sugar and fat, and a dark chocolate bar cannot be assumed to exert equivalent metabolic effects.

As Martín and colleagues emphasized in their analyses of cocoa and metabolic health, biological responses depend strongly on product formulation, the amount and bioavailability of flavanols delivered, and the metabolic characteristics of the study population [37,38]. For this reason, the present review focuses on cocoa-based plant matrices rather than isolated cocoa polyphenols.

Overall, current evidence indicates that cocoa polyphenols act as biologically relevant modulators of redox-sensitive pathways involved in glucose metabolism, although their effects are generally context-dependent and moderate rather than pronounced [36,37,38,39,40,41,42,43,44,45,46,47,48,49,50]. The strongest clinical evidence concerns endothelial function and nitric oxide-related signaling, while mechanistic and experimental studies suggest additional actions on oxidative stress, inflammatory signaling, GLUT4 translocation, and β-cell protection [37,38,39,41,43,44,45,46,47,48,49,50].

**Figure 2:**
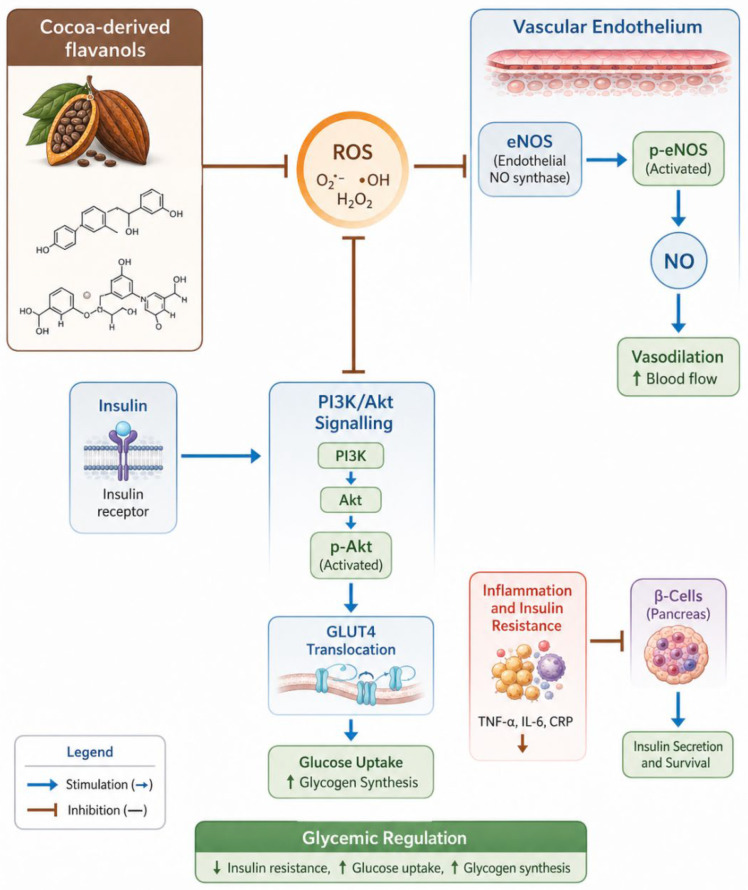
Mechanistic diagram of cocoa polyphenols and redox-sensitive pathways involved in glucose metabolism. Cocoa flavanols, particularly (−)-epicatechin, (+)-catechin, and procyanidins, may influence glucose homeostasis through modulation of redox-sensitive pathways related to endothelial nitric oxide bioavailability, PI3K/Akt and AMPK signaling, inflammatory tone, and tissue-specific glucose handling.

These mechanisms should not be considered as nonspecific antioxidant activity but as modulation of key processes linking metabolic overload with dysglycemia, including vascular responsiveness, intracellular insulin signaling, cellular redox balance, and postprandial metabolic stress [37,38,41,50]. Cocoa polyphenols therefore represent the most direct class of cocoa-derived bioactives linking redox biology with glucose metabolism, as seen in Figure 2. However, their metabolic effects cannot be understood independently of the surrounding food matrix. A cocoa product may contain substantial amounts of flavanols yet still exert unfavorable metabolic effects if delivered within a formulation that increases glycemic load or provides an adverse nutritional context.

### 4.7. Structure–Activity Relationships and Chemical Determinants of Biological Activity

Understanding the relationship between the chemical structure of cocoa flavanols and their biological activity is essential for interpreting the heterogeneity of available evidence and for designing more effective cocoa-based functional matrices. The main bioactive compounds in cocoa—(−)-epicatechin, (+)-catechin, and their oligomeric procyanidins—share the flavan-3-ol backbone but differ substantially in their spatial configuration, hydroxylation pattern, and degree of polymerization. These structural differences translate into differences in receptor binding, redox activity, bioavailability, and metabolic fate that must be considered when interpreting experimental findings.

The 3′,4′-dihydroxyl (catechol) moiety present in the B-ring of (−)-epicatechin is particularly relevant for biological activity. This ortho-dihydroxyl group confers strong metal-chelating capacity, facilitates hydrogen bonding interactions with biological macromolecules, and serves as the primary site for radical scavenging via single electron transfer. In the context of redox signaling, the catechol moiety may also interact with Keap1-Nrf2 complexes, facilitating nuclear translocation of Nrf2 and induction of antioxidant response element (ARE)-regulated genes such as heme oxygenase-1 (HO-1), NAD(P)H quinone oxidoreductase 1 (NQO1), and glutamate-cysteine ligase (GCL) [37,38]. These interactions help explain the apparent paradox that flavanols, despite being consumed at concentrations insufficient for significant direct antioxidant activity in biological fluids, can nonetheless produce measurable changes in cellular redox homeostasis—likely by activating endogenous antioxidant defense systems rather than by acting as exogenous radical scavengers.

Stereochemistry also plays an important role. (−)-Epicatechin (2R,3R configuration) and (+)-catechin (2R,3S configuration) are diastereomers that differ in the spatial orientation of the hydroxyl group at C-3. This difference affects their binding affinity to membrane lipids, plasma proteins, and enzyme active sites. (−)-Epicatechin predominates in fresh cocoa and is more bioavailable than its epimer; however, during thermal processing and alkalization, epicatechin undergoes epimerization, and additional degradation can occur, substantially altering the structural profile of the bioactive fraction [51,52]. This has direct implications for research interpretation: studies using standardized epicatechin preparations are not directly comparable to studies using commercially processed cocoa products with undefined epimerization profiles.

Procyanidins—oligomers formed by interflavan linkages between flavan-3-ol units, predominantly B-type (C4-C8 or C4-C6 linkages) in cocoa—exhibit distinct bioavailability and biological activity compared to monomers. Dimers (e.g., procyanidin B1, B2) are partially absorbed in the small intestine, while trimers and larger oligomers are poorly absorbed and reach the colon largely intact, where they serve as substrates for microbial biotransformation into phenolic metabolites with distinct biological activity [53]. Procyanidin oligomers with a higher degree of polymerization appear to exhibit stronger activation of AMPK-dependent glucose uptake pathways and pronounced effects on gut microbiota composition compared to monomers [38]. However, their markedly lower systemic bioavailability means that their principal biological effects may operate primarily through colonic and microbiota-mediated routes rather than through direct systemic action. These complementary structure–activity relationship (SAR) profiles suggest that the total flavanol content of a matrix is an insufficient descriptor of its biological potential; the distribution of oligomerization degrees and the processing conditions that determine their stability are equally important parameters. A conceptual scheme of the main cocoa bioactive structures and their proposed biological targets is presented in Figure 2 (Table 1).

**Table 1:** Cocoa-derived bioactive compounds and their proposed roles in glucose metabolism and redox signaling.

Bioactive Compound/Class	Source in Cocoa Matrix	Main Biological Target/Pathway	Proposed Metabolic Role	Evidence Type	Key Limitations
(−)-Epicatechin [28,29,30,38,44]	Dominant flavan-3-ol in cocoa	eNOS activation; PI3K/Akt; AMPK; NF-κB; Nrf2	Improved NO bioavailability; reduced oxidative stress; enhanced insulin signaling	Human RCT; preclinical; mechanistic	Dose variability; processing sensitivity; poor bioavailability of intact form
(+)-Catechin [38,44,53]	Cocoa beans; processing-labile	Redox signaling; anti-inflammatory; GLUT4 translocation	Antioxidant modulation; possible glycemic effects in animal models	Preclinical; in vitro; limited human data	Very limited direct human cocoa evidence; epimerizes to epicatechin during processing
Procyanidins (B1, B2; oligomers) [38,51,52,53]	Cocoa liquor; raw cocoa powder	AMPK; GLUT4 translocation; IGF-1R; insulin-independent glucose uptake	Improved glucose tolerance in rodent models; anti-inflammatory; microbiota modulation	Preclinical (mouse); in vitro; mechanistic; limited human	Trimers/tetramers poorly absorbed; most human evidence indirect from high-flavanol cocoa
Theobromine [25,26,27]	Methylxanthine; abundant in cocoa (1–2%)	Phosphodiesterase inhibition; vasodilation; weak AMPK activation	May improve vascular tone; limited direct glycemic evidence	In vitro; limited preclinical; very few human studies	Evidence predominantly extrapolated from xanthine biology; cocoa-specific human RCTs lacking
Caffeine [25,26,27]	Present at low amounts in cocoa (≤0.2%)	Adenosine receptor antagonism; lipolysis; insulinotropic at high dose	Modest acute glycemic and cardiovascular effects; context-dependent	Human studies (mixed populations); not specific to cocoa matrices	Very low concentration in cocoa; effects primarily from coffee literature
Phenolic acids (protocatechuic, ferulic) [53]	Cocoa shell; colonic metabolites of procyanidins	Nrf2/ARE; anti-inflammatory; microbiota biotransformation products	May contribute to antioxidant capacity after gut biotransformation	Mainly in vitro; colonic fermentation models; limited human	Largely produced by gut bacteria; bioavailability highly variable; cocoa-specific evidence sparse
Dietary fiber (cocoa shell) [31,32]	Cocoa pod husk; shell fractions	Gut microbiota modulation; SCFA production; intestinal barrier integrity	Prebiotic-like effects; reduced endotoxemia; possible glycemic modulation	Human feasibility studies; in vitro fermentation; limited RCT	Not present in standard chocolate; requires specific matrix inclusion; human glycemic evidence modest
Magnesium [54,55]	Cocoa powder (~500 mg/100 g); reduced in processing	Insulin receptor substrate activation; AMPK cofactor; antioxidant enzyme cofactor	Improved insulin sensitivity; reduced diabetes risk in deficient populations	Human cohort; meta-analysis; RCT (isolated supplementation)	Evidence mainly from supplementation studies, not cocoa-specific; modest effect sizes
Zinc [56]	Cocoa powder; cocoa nibs	Insulin synthesis/storage; SOD cofactor; Nrf2 activation	Supports β-cell function; antioxidant enzyme activity; glycemic control in deficiency	Meta-analysis (supplementation); mechanistic	Studies use zinc supplements, not cocoa; cocoa content modest; absorption depends on matrix

RCT, randomized controlled trial; eNOS, endothelial nitric oxide synthase; NO, nitric oxide; PI3K, phosphoinositide 3-kinase; Akt, protein kinase B; AMPK, AMP-activated protein kinase; GLUT4, glucose transporter type 4; SCFA, short-chain fatty acids; SOD, superoxide dismutase; Nrf2, nuclear factor erythroid 2-related factor 2. Evidence levels: in vitro (cell culture); preclinical (animal models); mechanistic (biochemical/molecular studies); human RCT (randomized controlled trial); meta-analysis (quantitative synthesis). Evidence for isolated procyanidins or high-flavanol cocoa preparations, not standard commercial chocolate (Table 2).

**Table 2:** Evidence strength across cocoa-based matrix components and extrapolated ingredients.

Component	Direct Cocoa-Based Human Evidence	Isolated Compound/Non-Cocoa Evidence	Preclinical/Mechanistic Evidence	Main Cardiometabolic Outcome	Overall Interpretation
Cocoa flavanols (whole dark chocolate) [28,29,30,33,34,35]	Moderate (human RCTs); consistent on endothelial markers	Extensive (isolated epicatechin; purified extracts)	Strong (eNOS, AMPK, GLUT4, anti-inflammatory)	Improved FMD; modest BP reduction; limited fasting glucose effects	Biologically plausible; endothelial effects more consistent than glycemic effects; matrix-dependent
Erythritol/polyols (in cocoa formulation) [57,58,59]	Limited (acute postprandial; T2D pilot)	Moderate (isolated studies)	Minimal	Reduced postprandial glycemia; improved endothelial markers (erythritol)	Glycemic load reduction rationale is strong; vascular data preliminary; long-term data scarce
Steviol glycosides (in cocoa matrix) [60,61,62]	None specific to cocoa matrices	Moderate (human preload studies)	Limited	Lower postprandial glucose vs. sucrose; no compensatory intake increase	Acute glycemic benefit well-supported; long-term evidence heterogeneous; not tested in cocoa
Inulin-type fructans (added to cocoa) [63,64,65]	Very limited	Moderate (isolated supplementation)	Strong (microbiota, SCFA, gut barrier)	Bifidogenesis; modest fasting glucose and HbA1c reduction in T2D/prediabetes (meta-analyses)	Mechanism plausible; benefits depend on dose, duration, host microbiota phenotype
Oat β-glucan (added to cocoa) [66,67,68,69]	None specific to cocoa	Strong (human RCTs with oat products)	Strong (viscosity; gastric emptying)	Reduced postprandial glycemia and LDL-cholesterol	Effects viscosity-dependent; processing may impair efficacy; no cocoa-specific trials
Oleic acid (oleic-rich cocoa formulation) [70,71,72,73,74,75,76]	None specific to cocoa matrices	Moderate (dietary pattern studies; MUFA substitution)	Moderate (membrane fluidity; insulin receptor function)	Improved insulin sensitivity; reduced postprandial lipemia; Mediterranean diet context	Benefits largely context-dependent; cocoa butter already contains oleic + stearic acid; studies use isolated fat substitutions
Magnesium (from cocoa) [54,55]	None specific to cocoa	Moderate-strong (supplementation meta-analyses)	Strong (insulin receptor; AMPK; antioxidant cofactor)	Improved insulin sensitivity; reduced T2D risk in deficient populations	Studies use supplements; cocoa contribution depends on processing; content decreases with alkalization

FMD, flow-mediated dilation; BP, blood pressure; RCT, randomized controlled trial; T2D, type 2 diabetes; HbA1c, glycated hemoglobin; LDL, low-density lipoprotein; MUFA, monounsaturated fatty acids; AMPK, AMP-activated protein kinase; eNOS, endothelial nitric oxide synthase; SCFA, short-chain fatty acids. Evidence classifications are based on the authors’ assessment of available literature at the time of manuscript preparation (Table 3).

**Table 3:** Non-glycemic sweeteners used in cocoa-based functional food matrices: metabolic characteristics and evidence summary.

Sweetener	Type	GI	Acute Postprandial Effect	Long-Term Evidence	Additional Effects	Key References
Erythritol	Polyol	0	Negligible glucose/insulin response; mainly excreted unchanged in urine	Heterogeneous; modest body weight reduction; improved endothelial function in T2D pilot	Improved small-vessel endothelial function; reduced central aortic stiffness indices	[77,78,79]
Xylitol/Sorbitol/Isomalt	Polyols	7–36	Markedly lower than sucrose; dose-dependent GI tolerance	Limited RCT data; generally safe for dental health; modest glycemic benefit	Dental health; lower postprandial insulin vs. sucrose	[80]
Steviol glycosides (Stevia)	High-intensity glycoside	<1	Lower postprandial glucose and insulin vs. sucrose; improved insulinogenic index in T2D	Limited long-term RCT; no compensatory energy intake reported in preload studies	May improve gut microbiota profile; not insulinotropic at recommended doses	[81,82]
Monk fruit (Lo Han Guo)	High-intensity glycoside	0	No significant increase in postprandial glucose vs. water or NNS controls	Very limited; extrapolated from beverage substitution studies	Antioxidant activity of mogrosides (preclinical); glycemic effect comparable to other NNS	[83]
Sucralose	Chlorinated sucrose derivative	0	Does not acutely raise blood glucose or insulin	12-wk trial in T2D Asians: no HbA1c change; modest anthropometric benefit	Stable at high temperature; compatible with chocolate processing	[57]
D-Allulose	Rare sugar (C3 epimer of fructose)	<1	Dose-dependent reduction in postprandial glucose/insulin; inhibits intestinal α-glucosidase	Limited; meta-analysis supports postprandial glycemia attenuation in healthy adults	Potential anti-obesity and hepatoprotective effects in animal models	[58,59]
Sucrose (reference)	Disaccharide (glucose + fructose)	65	Sharp postprandial glucose and insulin excursions; stimulates GLP-1 and GIP	Associated with cardiometabolic risk at high habitual intake	Provides flavor bulking but imposes glycemic burden	—

GI, glycemic index (relative to glucose = 100); NNS, non-nutritive sweeteners; T2D, type 2 diabetes; HbA1c, glycated hemoglobin; GLP-1, glucagon-like peptide 1; GIP, glucose-dependent insulinotropic polypeptide. GI values are approximate and may vary depending on food matrix context. All effects shown refer to substitution of sucrose unless stated otherwise (Table 4).

**Table 4:** Additive, complementary, and synergistic interactions among cocoa-based matrix components: conceptual framework and evidence status.

Interaction Type	Definition	Example in Cocoa-Based Formulations	Evidence Level	Interpretation
Additive	The combined effect equals the sum of individual effects; no positive or negative interaction between components	Cocoa flavanols (antioxidant effect) + magnesium (insulin cofactor) acting independently on separate metabolic targets	Mechanistic plausibility; no direct human evidence for the combination	Most interactions within cocoa-based matrices are likely additive at best; this is the conservative and scientifically defensible interpretation
Complementary (pathway-targeting)	Components act on different stages of the same pathway, producing a coordinated metabolic benefit without exceeding the sum of individual effects	Non-glycemic sweetener (reduces acute glycemic load) + viscous fiber (slows glucose absorption) + flavanols (improves endothelial responsiveness): each targets a different aspect of postprandial physiology	Mechanistic and indirect clinical evidence; no co-formulation RCT testing all three simultaneously	Strong mechanistic rationale; likely operative in well-designed cocoa matrices; still awaits direct experimental confirmation
Matrix-mediated modulation	One component alters the bioavailability, stability, or metabolic destination of another	Oleic-rich lipid fraction increases micellar solubilization and intestinal absorption of lipophilic flavanols; fiber creates a viscous environment that entraps polyphenols and modifies their release during digestion	Preclinical and in vitro evidence; some human bioaccessibility data; no direct cocoa-specific RCT	Biologically plausible and supported by food science; important for formulation optimization but not clinically confirmed for cocoa matrices
Kinetic synergy	Components reshape the temporal pattern of metabolic exposure rather than acting on the same molecular target, collectively reducing peak-to-trough variability	Viscous fiber (slows gastric emptying) + low-glycemic sweetener (displaces sucrose) + flavanols (attenuates postprandial oxidative stress): results in a flattened postprandial glucose curve with reduced oxidative burden	Mechanistic; extrapolated from individual component studies; not tested as a combined matrix intervention in humans	Relevant for reducing glycemic variability; direct evidence lacking
True synergy	The combined effect exceeds the sum of individual effects; requires dedicated factorial experimental design to confirm	Hypothetical: procyanidins + inulin-type fructans acting synergistically on gut microbiota to produce enhanced SCFA production and metabolic benefit beyond either component alone	Not currently demonstrated for cocoa-based matrices; design of confirmatory studies is a research priority	Cannot be claimed on current evidence; should be framed as a hypothesis requiring factorial crossover trials with standardized cocoa matrices

SCFA, short-chain fatty acids; RCT, randomized controlled trial. Evidence classifications reflect the authors’ assessment of available literature; interactions labeled as ‘true synergy’ require formal demonstration using factorial experimental designs that have not yet been conducted for cocoa-based functional food matrices.

## 5. Non-Glycemic Sweeteners and Postprandial Glycemic Control

Within cocoa-based functional matrices, non-glycemic sweeteners are of relevance principally as substitutes for sucrose in chocolate and other cocoa-derived products, where sucrose has historically dominated the formulation and where its replacement carries direct metabolic implications. Polyols (notably erythritol), high-intensity steviol glycosides, and rare sugars such as allulose and tagatose constitute the compound classes whose substitution for cocoa-product sucrose has been most extensively characterized mechanistically [57,58,59,77,78,79,80,81,82,83,84,85,86,87,88]; the discussion that follows examines this evidence in the framing that matters for cocoa-based reformulation.

Postprandial glycemic control represents a central nutritional target in metabolic disease, particularly in individuals with insulin resistance, prediabetes, and type 2 diabetes. Exaggerated and rapidly fluctuating postprandial glycemic responses contribute to oxidative stress, impair endothelial function, and contribute to progressive deterioration of insulin signaling and β-cell compensation [14,15,16]. Within this pathophysiological framework, the replacement of rapidly absorbable sugars with non-glycemic sweeteners is not merely a sensory reformulation strategy, but a metabolically meaningful intervention capable of modifying the acute glycemic burden of the food matrix. This issue is especially relevant for cocoa-based products, since conventional chocolate formulations often combine potentially beneficial cocoa flavanols with substantial amounts of sucrose. Accordingly, the scientific question is not simply whether sweeteners are “safer” than sugar in generic terms, but whether their inclusion in cocoa-based matrices can attenuate postprandial glycemic stress without undermining the broader metabolic rationale of the formulation [31,80].

### 5.1. Relevance for Nutritional Strategies and Transition Toward Cocoa-Based Matrices

The metabolic rationale for non-glycemic sweeteners is grounded in the kinetics of carbohydrate absorption and its impact on postprandial glycemia and insulinemia. Ludwig et al. emphasized that carbohydrate quality, not only carbohydrate quantity, influences chronic disease risk, particularly through differences in postprandial glycemia and insulinemia [31]. In practical terms, rapidly absorbed sugars such as glucose or sucrose induce sharp postprandial increases in plasma glucose, whereas sweeteners with negligible digestibility, minimal glycemic conversion, or very low caloric contribution can substantially blunt this response. This distinction is particularly important in food products designed for individuals with impaired glucose tolerance, as repeated attenuation of postprandial glycemic excursions may reduce downstream oxidative and vascular stress even in the absence of changes in fasting glycemia.

Among the best characterized non-glycemic sweeteners are polyols, especially erythritol. Livesey’s analysis of polyols as sugar replacers remains foundational because it quantified their glycemic and insulinemic behavior and showed that these compounds have markedly lower glycemic indices than conventional sugars [80]. Erythritol is essentially non-glycemic, whereas xylitol, sorbitol, and isomalt also exhibit low glycemic and insulinemic effects relative to sucrose [80]. These properties provide a metabolic basis for their use in reducing the glycemic load of food matrices.

Human studies indicate that erythritol is rapidly absorbed, largely unmetabolized, and excreted unchanged in urine, with negligible effects on circulating glucose and insulin concentrations [77,78]. This metabolic profile underlies its relevance in reformulation strategies, as it provides sweetness without inducing the acute glycemic and insulinemic responses associated with rapidly absorbable sugars [77,78].

Beyond glycemic neutrality, limited evidence suggests that erythritol may influence vascular-related outcomes. Flint et al. studied patients with type 2 diabetes and found that acute erythritol consumption improved small-vessel endothelial function, while chronic intake reduced central aortic stiffness indices [79]. Although this does not imply that erythritol is a vasoprotective therapy, it is a highly relevant observation for the present review because it suggests that at least some non-glycemic sweeteners may be metabolically compatible with vascular protection rather than simply metabolically inert [79]. Since endothelial dysfunction is one of the early consequences of postprandial oxidative stress, this observation provides a mechanistic rationale for combining low-glycemic sweeteners with cocoa polyphenols within functional food matrices [40,41,43,44].

Another important class of compounds includes high-intensity sweeteners such as steviol glycosides. In contrast to polyols, these substances are used at very low concentrations because of their intense sweetness, thereby contributing virtually no caloric or glycemic load. Gregersen et al. showed in patients with type 2 diabetes that adding stevioside to a standard meal reduced the incremental postprandial glucose response and increased the insulinogenic index, suggesting improved handling of the glucose challenge [81]. Anton et al., in a human preload study, found that Stevia produced lower postprandial glucose and insulin responses than sucrose and did so without compensatory increases in subsequent energy intake [82]. These studies are important because they indicate that certain non-glycemic sweeteners may exert favorable acute metabolic effects when they replace conventional sugars, rather than merely diluting total caloric exposure.

At the level of acute glycemic physiology, the broader human evidence is also informative. Greyling et al. conducted a systematic review and meta-analysis of randomized controlled trials examining the acute glycemic and insulinemic effects of low-energy sweeteners and concluded that they do not increase postprandial glucose or insulin relative to appropriate controls [84]. Nichol et al. reached a similar conclusion in their systematic synthesis of the glycemic impact of non-nutritive sweeteners, observing that, overall, their consumption did not elevate blood glucose in acute settings [81]. These findings are directly relevant to the present review because they support the formulation principle underpinning this section: replacing high-glycemic sugars with non-glycemic sweeteners can reduce the immediate glycemic burden of a cocoa-based food matrix.

This principle becomes especially important when cocoa is considered as a complete food matrix rather than an isolated flavanol source. A cocoa matrix enriched in flavanols may still be metabolically suboptimal if it delivers a high sucrose load. Conversely, a cocoa-based product reformulated with non-glycemic sweeteners may preserve the redox- and vascular-related benefits associated with cocoa polyphenols while reducing the acute glycemic stress imposed by the carbohydrate fraction. In this sense, sweetener choice is not merely a technological consideration but a key determinant of the metabolic properties of the matrix.

Recent evidence has also broadened the range of sugar alternatives under discussion. D-allulose, a rare sugar with negligible energetic contribution, has attracted increasing interest because of its ability to attenuate postprandial glycemia while preserving sweetness. Tani et al., in a systematic review and meta-analysis of human studies, concluded that D-allulose significantly attenuates postprandial blood glucose concentrations in healthy individuals [58]. More recently, Buranapin et al. showed in a randomized crossover trial that adding D-allulose to a sucrose beverage produced dose-dependent reductions in postprandial glucose and insulin responses in healthy volunteers [59]. Although D-allulose occupies a somewhat different regulatory and technological niche from polyols or classic high-intensity sweeteners, these findings reinforce the broader concept that sugar replacement strategies can meaningfully modulate acute glycemic physiology.

### 5.2. Human Evidence: Promise, Inconsistency, and Context Dependence

Despite the strong physiological rationale and several favorable intervention studies, the human literature on non-glycemic sweeteners remains heterogeneous and requires careful interpretation. This heterogeneity arises partly from the fact that different compounds behave differently and partly because study designs vary substantially with respect to duration, comparator, delivery vehicle, and participant phenotype. In acute settings, the evidence is relatively consistent: compared with sucrose or glucose, non-glycemic sweeteners tend to produce lower postprandial glycemic and insulinemic responses. However, when the outcomes shift toward longer-term endpoints such as HbA1c, body weight, insulin sensitivity, or cardiometabolic risk, the evidence becomes more mixed.

Tey et al. examined beverages sweetened with aspartame, monk fruit, stevia, or sucrose and found that, although sucrose caused sharper early glucose and insulin excursions, the overall 3 h glucose and insulin area under the curve did not differ significantly across beverages when subsequent intake was considered [83]. In a related study, the same group reported that replacing a single sucrose-sweetened beverage with non-nutritive sweetened alternatives had minimal effects on 24 h glucose profiles in healthy men [88]. These observations highlight a key limitation: acute glycemic neutrality does not necessarily translate into meaningful improvements in whole-day metabolic regulation [83,88].

This theme is reinforced by the network meta-analysis of Zhang et al., who concluded that non-nutritive sweetened beverages generally have no acute metabolic or endocrine effects beyond those seen with water, while calorically sweetened beverages increase postprandial glucose, insulin, GLP-1, and GIP responses [85]. In other words, the clearest benefit of non-glycemic sweeteners may not lie in producing active metabolic enhancement, but in avoiding the metabolic perturbation induced by rapidly absorbable sugars [85]. For a narrative review focused on cocoa-based matrices, this is a crucial distinction. The metabolic value of a sweetener is often substitutional rather than intrinsically pharmacological.

This substitution-based interpretation is further supported by longer-term evidence, which remains heterogeneous and of limited certainty. Toews et al., in a broad systematic review and meta-analysis, found limited evidence of meaningful long-term health benefits or harms from non-sugar sweeteners, largely because of heterogeneity and low-certainty evidence across studies [86]. Azad et al. likewise reported that randomized controlled trials did not consistently support major benefits of nonnutritive sweeteners on adiposity or cardiometabolic outcomes, whereas observational cohorts often showed associations with higher cardiometabolic risk, likely influenced by confounding and reverse causality [87]. This discrepancy between trial evidence and observational associations is particularly important. Individuals at higher metabolic risk are more likely to choose diet products, making it difficult to infer causality from habitual consumption patterns alone [86,87].

Evidence, specifically in individuals with diabetes, remains comparatively limited. In a Cochrane review focused on non-nutritive sweeteners in diabetes mellitus, Lohner et al. concluded that the available randomized evidence was insufficient to support robust long-term claims regarding glycemic improvement, largely because of small sample sizes, short intervention periods, and substantial methodological heterogeneity across studies [88]. This reinforces a consistent pattern in the literature: while the acute postprandial neutrality of many non-glycemic sweeteners is well established, their capacity to induce sustained improvements in established diabetic populations remains uncertain.

More recently, Mohan et al. evaluated the replacement of sucrose with sucralose in tea and coffee over 12 weeks in Asian Indians with type 2 diabetes and found no significant changes in HbA1c or fasting plasma glucose, although modest reductions in body weight, body mass index, and waist circumference were observed [57]. These findings are informative because they suggest that sugar replacement may improve certain anthropometric or dietary markers without necessarily producing large short-term changes in conventional glycemic endpoints when pharmacological treatment and habitual dietary patterns are already established [57]. Thus, the metabolic value of non-glycemic sweeteners in diabetes may depend less on direct glucose-lowering effects and more on their role in reducing cumulative exposure to rapidly absorbable sugars over time.

Additional evidence from emerging sugar alternatives also supports the concept that formulation choices can meaningfully alter acute glycemic physiology. Tani et al., in a systematic review and meta-analysis of human studies, concluded that D-allulose significantly attenuates postprandial blood glucose concentrations in healthy participants [58]. In line with this, Buranapin et al. showed in a randomized crossover trial that adding D-allulose to a sucrose-containing beverage reduced postprandial glucose and insulin responses in a dose-dependent manner in healthy Thai volunteers [59]. Although these studies were conducted outside the specific context of cocoa-based foods, they remain highly relevant for the present review because they strengthen the broader formulation principle that replacing or partially displacing conventional sugars with low-glycemic sweetening agents can reduce acute metabolic stress.

These inconsistencies do not negate the relevance of non-glycemic sweeteners but rather define the conditions under which their effects are most meaningful. Their principal utility lies in reducing the glycemic load of the matrix and attenuating acute postprandial stress, not necessarily in acting as stand-alone antidiabetic agents. This interpretation is fully compatible with the matrix-based approach adopted throughout the manuscript. This interpretation aligns with a matrix-based perspective, in which non-glycemic sweeteners function as supportive formulation tools that help preserve the redox- and vascular-related effects of cocoa polyphenols by limiting sucrose-driven glycemic excursions.

This context-dependent interpretation is especially important because the effects of sweeteners cannot be disentangled from the physical and compositional characteristics of the product in which they are delivered. A cocoa beverage sweetened with erythritol or stevia, a dark chocolate bar reformulated with a polyol blend, and a low-sugar cocoa dessert containing additional fibers will not generate identical digestive or metabolic responses. The final postprandial profile depends on the combination of sweetness profile, carbohydrate displacement, food structure, fat content, and accompanying bioactive compounds. For this reason, the role of non-glycemic sweeteners in cocoa-based matrices should be interpreted in formulation terms, not merely ingredient terms.

Taken together, current evidence suggests that non-glycemic sweeteners are metabolically useful primarily because they reduce exposure to rapidly absorbable sugars and attenuate postprandial glycemic stress [31,77,78,79,80,81,82,83,84,85]. Human intervention studies provide the strongest support for neutral or favorable acute postprandial effects, particularly for erythritol, steviol glycosides, and other low-energy sweeteners used in substitution contexts [79,81,82,83,84,85]. At the same time, long-term outcomes remain more heterogeneous, with systematic reviews highlighting modest benefits at best and substantial context dependence [57,86,87,88]. Thus, the main translational implication is clear: when cocoa is formulated as a functional matrix for metabolic health, the choice of sweetener becomes a decisive determinant of whether the final product attenuates or amplifies postprandial glycemic burden.

Emerging cardiovascular evidence has nevertheless qualified the erythritol safety profile. Witkowski et al. reported that circulating erythritol concentrations were associated with major adverse cardiovascular events in two large observational cohorts and demonstrated, in human and preclinical experiments, that erythritol enhances platelet aggregation and thrombosis potential [89,90]. These findings do not establish causality but indicate that the cardiovascular safety of repeated high-dose erythritol exposure cannot be assumed neutral, and they argue for moderate replacement strategies in cocoa-product reformulation—and for combining erythritol with steviol glycosides or rare sugars rather than relying on high single-sweetener doses—pending adequately powered prospective trials.

## 6. Prebiotic Fibers and Microbiota-Mediated Metabolic Effects

Dietary fiber is a major modulator of postprandial glycemia and microbiota-derived short-chain fatty acid production, with the recommended daily intake for adults of approximately 25–30 g·day^−1^. In a cocoa formulation context, the absolute fiber contribution depends on serving size. Cocoa powder contains approximately 10–12 g of total fiber per 100 g, of which roughly one third is soluble, so a typical 10 g serving contributes ~1 g—small in isolation, yet biologically relevant when delivered alongside the polyphenol, lipid and protein components of the cocoa matrix [53,89,90]. This quantitative anchoring frames the mechanistic and clinical evidence that follows.

The current conceptual basis for prebiotics was formalized by the International Scientific Association for Probiotics and Prebiotics, which defined a prebiotic as “a substrate that is selectively utilized by host microorganisms conferring a health benefit” [60]. This definition is especially relevant for a matrix-based review because it emphasizes that not all dietary fibers are prebiotics and that the defining feature is not merely fermentability, but selective microbial utilization linked to a measurable benefit for the host [60]. In metabolic disease, this benefit is increasingly interpreted through pathways involving microbial composition, short-chain fatty acid generation, modulation of gut-derived satiety and incretin hormones, reduction of endotoxemia, and attenuation of low-grade inflammation [32,60,62].

Among the prebiotic substrates most consistently studied in relation to metabolic health are inulin-type fructans, including inulin and oligofructose. Hughes et al., in a systematic review of human clinical studies, concluded that inulin-type fructans reliably exert bifidogenic effects and may also improve intestinal barrier function, triglycerides, satiety, and, in some contexts, insulin sensitivity [61]. Although the magnitude of these effects varies according to chain length, dose, duration, and host phenotype, this review remains highly relevant because it shows that the metabolic significance of these fibers cannot be reduced to bowel function alone; rather, they may influence several metabolic endpoints through microbiota-mediated mechanisms [61].

Canfora et al. further strengthened this mechanistic framework by placing prebiotic fermentation within the broader biology of obesity, insulin resistance, NAFLD, and type 2 diabetes [62]. Their work highlighted that indigestible carbohydrates are converted by the microbiota into metabolites such as acetate, propionate, and butyrate, which may affect host physiology via G-protein-coupled receptors, intestinal gluconeogenesis, adipose tissue signaling, and systemic inflammatory pathways [62]. From the perspective of the present review, this is a key transition: prebiotic fibers are relevant not because they are “healthy fibers” in a generic sense, but because they generate microbial metabolites that can participate in pathways directly linked to glucose homeostasis and metabolic resilience.

### 6.1. Relevance for Nutritional Strategies and Transition Toward Cocoa-Based Matrices

The metabolic relevance of prebiotic fibers becomes clearer when one considers the multiple interfaces between the gut microbiota and host glucose regulation. Experimental and translational studies have repeatedly suggested that fermentable fibers can influence satiety, insulin sensitivity, endotoxemia, and gut hormone secretion. Cani et al. were among the first to translate this concept into humans, showing in a pilot study that oligofructose supplementation promoted satiety and reduced hunger-related responses in healthy subjects [91]. Although this was not a diabetes study, it was mechanistically important because it suggested that fermentable fibers may influence host metabolic regulation through gut-derived signaling rather than simply through caloric dilution [91].

This concept was subsequently expanded by Parnell and Reimer, who reported that 12 weeks of oligofructose supplementation in overweight and obese adults was associated with modest weight loss and modulated appetite-related hormones, including lower ghrelin and higher peptide YY concentrations [92]. Although these effects do not demonstrate a direct antidiabetic action, they support the concept that prebiotic fibers influence metabolic risk through endocrine pathways regulating food intake and energy balance.

In populations at elevated metabolic risk, the evidence becomes more clinically relevant. Guess et al. conducted a randomized crossover trial in individuals with prediabetes and found that inulin may have unique metabolic effects in specific prediabetic phenotypes, although the overall effects on fasting glucose and insulin sensitivity were modest [93]. This pattern highlights a recurring feature of microbiota-targeted interventions: effects are often phenotype-dependent, and improvements may be more apparent in selected metabolic subgroups than in broad heterogeneous cohorts [93].

A similar theme emerged in the randomized controlled trial by Dehghan et al. in women with type 2 diabetes. These authors reported that oligofructose-enriched inulin improved some inflammatory markers and reduced metabolic endotoxemia, with parallel favorable changes in fasting glucose and HbA1c [94]. These findings suggest that the metabolic effects of prebiotic fibers extend beyond glycemic control and involve interconnected pathways related to intestinal barrier function, endotoxin-related signaling, and systemic inflammation. Accordingly, their clinical relevance may not be fully captured by glucose-based endpoints alone but should be interpreted within a broader framework of microbiota-mediated metabolic regulation.

This broader interpretation is supported by the work of Chambers et al., who studied adults with overweight and obesity and found that both inulin-propionate ester and inulin improved insulin sensitivity compared with cellulose, albeit with distinct effects on the gut microbiota, plasma metabolome, and inflammatory responses [95]. This trial is especially informative because it moves beyond generic “fiber effects” and shows that even among fermentable substrates, microbiota-targeted outcomes may differ according to the structure and metabolic destination of the fiber [95]. The study also supports an important translational concept for the present review: when designing a functional cocoa-based matrix, the type of fermentable substrate matters, not only its quantity.

The most comprehensive synthesis of this evidence was provided by Wang et al., who conducted a GRADE-assessed systematic review and dose–response meta-analysis of 33 randomized controlled trials and concluded that inulin-type fructan supplementation significantly improved fasting glucose, HbA1c, fasting insulin, and HOMA-IR, particularly in prediabetes and type 2 diabetes [96]. This meta-analysis is central to this section because it indicates that prebiotic fibers may exert metabolically meaningful effects in dysglycemic populations, especially when administered at sufficient dose and duration [96]. However, it also reinforces the need for nuance: the effect size is not uniform, and the response appears to depend on population characteristics, intervention length, and the specific fructan formulation used [96].

At the level of the cocoa matrix, these findings are highly relevant. Cocoa-based formulations can incorporate fermentable fibers directly, or they may contain fiber-rich ingredients derived from cocoa byproducts such as cocoa shell fractions. In such contexts, the role of prebiotic fibers is not limited to slowing digestion (an issue that will be addressed more specifically in Section 6 for β-glucans and soluble fibers) but extends to shaping the downstream microbial metabolism of the matrix. This distinction is important to avoid redundancy across sections. Here, the focus is not viscosity or carbohydrate entrapment, but microbial selectivity, fermentation, and host–microbiota signaling.

Cocoa itself offers a compelling bridge between these concepts. Tzounis et al. demonstrated in a randomized, controlled, double-blind crossover trial that high cocoa flavanol consumption increased fecal bifidobacteria and lactobacilli and reduced clostridia in healthy humans, while concomitantly lowering plasma triglycerides and C-reactive protein [89]. These findings suggest that cocoa-derived components can exert microbiota-modulating effects in vivo, even in the absence of isolated fiber supplementation [89]. Sorrenti et al. later synthesized the bidirectional interaction between cocoa polyphenols and the microbiota, emphasizing that cocoa constituents may exert prebiotic-like effects while also being transformed into smaller metabolites with distinct biological activity [97]. Thus, in cocoa-based matrices, polyphenols and fermentable fibers should not be conceptualized as independent domains; they may converge in the colon and generate overlapping microbiota-mediated effects [89,97].

The same matrix-oriented reasoning applies to cocoa fiber itself. Sarriá et al. reported that regular consumption of soluble fiber-rich cocoa products improved bowel habits in healthy adults and increased fiber intake to recommended levels [90]. Although the study did not directly evaluate microbiota composition, it remains relevant because it supports the feasibility of using cocoa-derived fiber as a functional ingredient with physiological effects in humans [90]. More recently, Cañas et al. showed that phenolic compounds from cocoa shell flour and cocoa shell extract underwent extensive biotransformation during in vitro colonic fermentation, generating phenyl-γ-valerolactones and related microbial metabolites [53]. This work is mechanistically significant because it suggests that cocoa shell-derived matrices may provide not only fiber but also colon-delivered substrates that participate in microbiota-driven metabolic transformations [53].

### 6.2. Human Evidence: Promise, Inconsistency, and Context Dependence

Despite the strong mechanistic rationale supporting prebiotic fibers, the human evidence remains context-dependent and should not be overstated. Mitchell et al., in a pilot randomized controlled trial in adults at elevated risk for type 2 diabetes, found that inulin supplementation increased bifidobacteria but did not improve peripheral insulin sensitivity [98]. This dissociation highlights a key limitation: modulation of microbiota composition does not automatically translate into measurable improvements in glycemic physiology, particularly over short intervention periods. It also suggests that microbial function, metabolite production, and host metabolic context are critical determinants of clinical outcomes. Across the literature, beneficial effects of prebiotic fibers are more consistently observed for intermediate outcomes, including bifidogenicity, satiety-related hormones, and inflammatory markers, than for robust clinical endpoints such as HbA1c or long-term insulin sensitivity [61,93,98]. In other words, human evidence supports promise, but not uniformity. This is particularly relevant for a narrative review that aims to remain mechanistically rigorous rather than promotional. Prebiotic fibers should not be presented as direct antidiabetic agents in the pharmaceutical sense. Rather, they should be interpreted as matrix components that may improve the metabolic milieu through microbiota-mediated routes whose magnitude depends on baseline dysbiosis, dietary pattern, dose, fermentability, and host metabolic status [61,62,96].

These contextual dependencies are especially important in relation to cocoa-based matrices. A cocoa product enriched with fermentable fibers may not generate identical effects to isolated inulin supplementation, because the final response will depend on the co-presence of polyphenols, lipids, sweeteners, and the physical structure of the food. In this sense, microbiota-mediated metabolic effects are intrinsically matrix-dependent. The same fermentable substrate may produce different outcomes when delivered in a cocoa beverage, a reformulated chocolate, or a fiber-enriched cocoa snack, owing to differences in digestibility, substrate accessibility, colonic delivery, and interaction with other compounds. This reinforces one of the central arguments of the present review: the metabolic potential of cocoa-based foods emerges from the organization and interaction of components within the matrix, not from a single ingredient considered in isolation.

Taken together, the current body of evidence supports the view that prebiotic fibers are relevant to metabolic health because they influence the gut ecosystem and its signaling interface with the host [53,60,61,62,89,90,91,92,93,94,95,96,97,98]. Human and translational studies indicate that fermentable fibers can modulate satiety-related peptides, inflammatory tone, endotoxemia, and, in some contexts, glycemic control and insulin sensitivity [91,92,93,94,95,96,98]. Cocoa-related evidence further suggests that cocoa constituents themselves may participate in microbiota modulation and colonic biotransformation, thereby reinforcing the plausibility of cocoa-based matrices as vehicles for microbiota-mediated metabolic effects [53,89,90,97]. However, the magnitude of these responses remains variable and strongly context-dependent. Prebiotic fibers should therefore not be interpreted as independent antidiabetic agents, but as components that can enhance the metabolic quality of food matrices, shifting part of the physiological response from rapid carbohydrate exposure toward slower, microbiota-mediated, and potentially more resilient metabolic signaling. This provides the conceptual bridge to the next section, in which β-glucans and soluble fibers will be examined from a complementary perspective, focusing more specifically on their physicochemical effects on glycemic and lipid regulation.

## 7. β-Glucans and Soluble Fibers in Glycemic and Lipid Regulation

β-Glucans are not endogenous constituents of cocoa; they are characteristic soluble polysaccharides of oat and barley endosperm cell walls and of yeast cell walls. In a cocoa-formulation context, they therefore enter the discussion as candidate co-formulation ingredients rather than as native cocoa components, with the rationale for their incorporation derived from the robust and quantitatively consistent body of evidence on viscous β-glucan-driven attenuation of postprandial glycemic and lipid responses at clinically relevant doses (≥3 g per meal, molecular weight ≥200 kDa) developed below.

Prebiotic fibers, whose metabolic relevance depends largely on selective microbial utilization and downstream fermentation products, β-glucans and other hand, are viscous soluble fibers which exert a substantial part of their physiological effect within the upper gastrointestinal tract. Their importance in metabolic regulation derives primarily from physicochemical properties such as viscosity, gel formation, delayed gastric emptying, and reduced diffusion of nutrients toward the absorptive surface of the small intestine. These effects make β-glucans and related soluble fibers particularly relevant to the present review, because they can influence both acute postprandial glycemic responses and longer-term lipid metabolism without requiring a direct pharmacological action on host cells [99,100,101,102]. In the context of cocoa-based plant matrices, this distinction is central. Whereas non-glycemic sweeteners reduce glycemic burden by replacing rapidly absorbable sugars, viscous fibers modify the kinetics of nutrient delivery and absorption, thereby reshaping the metabolic meaning of the matrix itself.

β-Glucans are non-starch polysaccharides found principally in oats and barley and are among the most extensively studied soluble fibers in cardiometabolic nutrition. Their capacity to lower postprandial glycemia and circulating LDL-cholesterol has been recognized for decades, but more recent work has clarified that these effects depend not merely on total fiber content, but on structural features such as molecular weight, extractability, and the viscosity generated within the intestinal lumen [99,100,101,102]. This is especially important for a matrix-oriented review, because the physiological behavior of β-glucans is inseparable from processing, food structure, and co-ingested nutrients. A low-viscosity or highly degraded β-glucan preparation may not produce the same metabolic effect as a structurally preserved oat or barley matrix, even if nominal fiber content is similar [100,102].

The acute glycemic effects of oat β-glucan have been examined in considerable detail. Zurbau et al., in a systematic review and meta-analysis, concluded that oat β-glucan significantly reduced postprandial blood glucose and insulin responses, with the magnitude of the effect depending strongly on the dose and viscosity of the β-glucan delivered [99]. This finding is highly relevant because it confirms that β-glucans are not simply inert bulking agents; rather, they actively alter the digestive and absorptive handling of carbohydrates [99]. Wolever et al. extended this mechanistic interpretation in a randomized placebo-controlled crossover trial in healthy adults, showing that increasing β-glucan viscosity within a breakfast meal slowed gastric emptying and reduced glycemic and insulinemic responses, even though appetite-related hormones and subsequent food intake were not significantly altered [100]. This study is especially informative because it links a measurable physicochemical property—viscosity—to a defined metabolic outcome, thereby reinforcing the mechanistic plausibility of β-glucan-enriched matrices [100].

Earlier human trials reached similar conclusions. Hlebowicz et al. reported that a muesli product containing 4 g of oat β-glucan lowered postprandial blood glucose compared with cornflakes in healthy subjects, although it did not significantly alter gastric emptying or subjective satiety [101]. Panahi et al. later showed that β-glucan concentrates derived from oats differed in their glycemic effects according to the viscosity retained after processing, demonstrating that the preservation of rheological functionality is crucial for postprandial control [102]. Taken together, these studies support a consistent interpretation: the acute glycemic benefit of β-glucans depends not only on the presence of soluble fiber per se, but on whether the matrix preserves the physicochemical conditions required for viscous behavior in vivo [100,101,102].

The same principle applies to other viscous soluble fibers. Williams et al. showed that incorporation of guar gum and alginate into a cereal bar improved postprandial glycemia in humans, indicating that gel-forming soluble fibers can influence acute carbohydrate handling even when delivered in processed snack formats [63]. In patients with type 2 diabetes, de Carvalho et al. compared breakfasts containing soluble fiber from foods with breakfasts containing guar gum supplements and found that both approaches improved postprandial plasma glucose compared with a low-fiber control, suggesting that the source and delivery format of soluble fiber may matter less than the presence of sufficient viscous soluble fiber within the meal [64]. This concept is particularly relevant for cocoa-based products, as incorporation of soluble fibers into chocolate or cocoa-containing matrices may influence acute glycemic physiology, provided that the final structure preserves the functional properties required for viscosity.

### 7.1. Relevance for Nutritional Strategies and Transition Toward Cocoa-Based Matrices

From a translational standpoint, the relevance of β-glucans and viscous soluble fibers lies in their dual action on glucose and lipid metabolism. In glycemic terms, their capacity to increase intraluminal viscosity slows gastric emptying, delays starch hydrolysis, reduces glucose diffusion, and attenuates the rate of intestinal absorption [63,64,99,100,101,102]. In lipid terms, soluble fibers can interfere with bile acid reabsorption, increase fecal sterol loss, and promote compensatory hepatic cholesterol utilization, thereby contributing to reductions in circulating LDL-cholesterol [68,69,103,104]. This dual mechanism is particularly relevant in populations with overlapping dysglycemia and dyslipidemia, such as those with metabolic syndrome, prediabetes, or T2D.

Evidence for the glycemic effects of viscous fibers extends beyond β-glucans. Gibb et al., in a meta-analysis of psyllium supplementation, reported that psyllium improved glycemic control in proportion to the degree of pre-existing dysglycemia, with more pronounced effects in individuals with T2D than in normoglycemic participants [65]. More recently, Gholami et al. performed a GRADE-assessed meta-analysis and found that psyllium significantly reduced fasting blood sugar, HbA1c, and HOMA-IR across randomized controlled trials [66]. These studies are important because they indicate that soluble fibers can produce clinically relevant glycemic effects not only acutely but also over longer intervention periods, particularly in metabolically compromised populations [65,66]. Lu et al. further broadened this evidence base by showing, in a meta-analysis of randomized clinical trials, that viscous soluble dietary fiber supplementation improved both glucose and lipid metabolism in patients with type 2 diabetes [67]. Thus, the metabolic relevance of soluble fibers is not restricted to oats and barley, although β-glucans remain the most standardized and best characterized compounds in this category.

The lipid-lowering evidence for β-glucans is even more established. Whitehead et al. conducted a meta-analysis of randomized controlled trials and showed that consuming at least 3 g/day of oat β-glucan significantly reduced total and LDL-cholesterol concentrations [68]. Ho et al. later extended this observation, demonstrating that oat β-glucan reduced LDL-cholesterol, non-HDL-cholesterol, and apolipoprotein B, thereby strengthening the cardiovascular relevance of β-glucan-containing matrices [69]. Comparable results were reported for barley β-glucan by AbuMweis et al., who found significant reductions in total and LDL-cholesterol across randomized trials [103]. More recently, Yu et al. confirmed that oat β-glucan intake significantly decreased total and LDL-cholesterol in hypercholesterolemia adults, although effects on triglycerides and HDL-cholesterol were not consistent [104]. These studies are particularly important for the present review because they demonstrate that β-glucans can affect not only acute glycemic handling but also chronic lipid-related risk markers, thereby increasing the translational value of formulations that incorporate them [68,69,103,104].

At the level of food design, these findings have direct implications for cocoa-based matrices. Cocoa-derived foods intended for metabolic health are often conceptualized primarily around flavanol content, but the present section shows that soluble fibers may shape the metabolic response just as decisively. A cocoa product enriched with β-glucans or other viscous soluble fibers may attenuate postprandial glycemia through slower carbohydrate delivery while simultaneously contributing to LDL-cholesterol lowering over repeated consumption. This dual action is particularly relevant in energy-dense cocoa matrices that may contain added sugars, as inclusion of functional soluble fibers can partially counterbalance these features and improve the overall metabolic profile of the product.

Importantly, the final effect will depend on the way β-glucans are delivered. Xu et al., in a meta-analysis of mildly hypercholesterolemic individuals, reported that β-glucan retained cholesterol-lowering efficacy across different delivery matrices, although some solid–liquid integrated formats appeared more effective than others [105]. This observation is highly relevant for the present review because it reinforces the broader thesis that the metabolic effects of bioactive compounds are matrix dependent. A β-glucan-fortified cocoa beverage, a fiber-enriched chocolate bar, and a spoonable cocoa dessert will not necessarily produce identical glycemic or lipid responses, even if the nominal β-glucan dose is similar. Processing, water availability, particle structure, and co-ingredients all affect viscosity generation and intestinal behavior [100,102,105].

### 7.2. Human Evidence: Promise, Inconsistency, and Context Dependence

Although the overall human evidence for β-glucans and soluble fibers is favorable, it is not entirely uniform. One reason is that acute postprandial effects are generally easier to detect than longer-term changes in glycemic control. In acute crossover studies, viscous fibers often show clear reductions in postprandial glucose and insulin responses [63,64,99,100,101,102]. However, longer-term outcomes such as HbA1c, fasting insulin, or body weight are influenced by multiple factors, including baseline metabolic status, habitual diet, fiber dose, molecular characteristics, and treatment adherence. This helps explain why some interventions report robust postprandial effects but only modest changes in chronic biomarkers.

The evidence also indicates that not all soluble fibers behave identically. β-Glucans, psyllium, guar gum, and alginate differ in fermentability, viscosity, processing stability, and sensory compatibility. Moreover, even within β-glucans, molecular weight and degradation during processing can profoundly affect physiological efficacy [99,100,101,102]. Thus, the category “soluble fiber” is mechanistically useful but nutritionally insufficient if taken as a uniform entity. For a narrative review centered on food matrices, this point is essential: one cannot assume that adding any soluble fiber to a cocoa-based formulation will automatically produce meaningful metabolic benefit. The nature of the fiber and the conditions under which it is delivered must be considered explicitly.

An additional source of variability relates to disease phenotype, as the metabolic effects of viscous soluble fibers are more pronounced in individuals with impaired glucose regulation or dyslipidemia than in metabolically healthy populations [65,67]. This suggests that the metabolic relevance of soluble fiber enrichment may be greatest in populations already characterized by impaired glycemic control, postprandial dysmetabolism, or dyslipidemia.

Such variability does not undermine the relevance of β-glucans and soluble fibers but helps define their role. These compounds should not be understood as independent antidiabetic agents acting through a single molecular target. Their value lies in modifying digestive physiology, improving postprandial glycemic handling, and, in some cases, lowering LDL-related lipid risk over time [63,64,65,66,67,68,69,99,100,101,102,103,104,105]. In cocoa-based matrices, this means that soluble fibers may serve as structural metabolic modulators that complement the redox- and endothelial-related actions of cocoa flavanols and the glycemic load reduction achieved by non-glycemic sweeteners.

Taken together, the available evidence indicates that β-glucans and other viscous soluble fibers are among the most useful matrix-level tools for improving postprandial glycemic regulation and selected lipid markers [63,64,65,66,67,68,69,99,100,101,102,103,104,105]. Human trials and meta-analyses consistently support acute reductions in postprandial glucose and insulin, especially when viscosity is preserved, while longer-term studies indicate favorable effects on LDL-cholesterol and, in some contexts, glycemic control [65,66,67,68,69,103,104,105]. Within cocoa-based plant matrices, these fibers may therefore enhance metabolic functionality by reducing glycemic excursion, moderating digestive kinetics, and improving lipid-related risk markers.

## 8. Oleic-Rich Lipid Matrices and Insulin Sensitivity

The lipid fraction of cocoa butter is dominated by saturated fatty acids—principally palmitic acid (~25–30%) and stearic acid (~33–35%)—together with monounsaturated oleic acid (~33–35%). The metabolic profile of cocoa-based foods can therefore be modulated by partial substitution of the saturated fraction with high-oleic lipid sources, and this substitution constitutes the central question that motivates the present section. The broader Mediterranean pattern and oleic acid literature is extensively synthesized elsewhere and is invoked here only where it directly informs the cocoa butter substitution argument.

Oleic-rich lipid matrices present a metabolic relevance which depends neither primarily on direct radical scavenging, as in the case of cocoa polyphenols, nor on fermentation-mediated host–microbiota interactions, as discussed for prebiotic fibers, nor on viscosity-driven modulation of nutrient delivery, as described for β-glucans and soluble fibers. Rather, their significance lies in the way they alter lipid quality within the matrix and, through this, influence membrane dynamics, intracellular lipid partitioning, postprandial lipemia, inflammatory signaling, and insulin action. This distinction is especially important in the context of cocoa-based formulations. Cocoa products are frequently judged according to sugar content or polyphenol concentration, yet the lipid architecture of the matrix can also shape the final metabolic response, particularly when oleic-rich ingredients are incorporated as part of reformulation strategies aimed at improving cardiometabolic quality [70,71,72,73].

From a physiological standpoint, the effects of dietary fat on insulin sensitivity depend not only on the total amount of fat consumed but also on fatty acid composition, food structure, and the nutrients displaced by that fat within the diet. Substituting saturated fats or rapidly absorbable carbohydrates with monounsaturated fatty acids (MUFA), particularly oleic acid, may improve insulin sensitivity and glycemic regulation under certain conditions, especially in individuals with insulin resistance, central adiposity, or features of the metabolic syndrome [70,71,74]. This is relevant to the present review because oleic-rich matrices are not being considered here as isolated lipid supplements, but as structural components of functional foods that may modulate the overall metabolic meaning of the cocoa matrix.

Oleic acid is the predominant fatty acid in olive oil and is also found in significant amounts in nuts, avocado, and selected lipid blends used in food formulation. Its metabolic effects have traditionally been interpreted within the broader framework of Mediterranean dietary patterns, but a more mechanistic literature has progressively clarified that oleic-rich matrices may influence insulin sensitivity through several overlapping routes, including altered membrane fluidity, improved insulin receptor function, reduced generation of lipotoxic intermediates, attenuation of inflammatory signaling, and modulation of postprandial lipid handling [70,71,72,73,74,75,76]. The metabolic relevance of oleic-rich matrices therefore lies not in their classification as “healthy fats,” but in their capacity to replace less favorable lipid or carbohydrate profiles and to reshape the overall metabolic behavior of the food matrix.

### 8.1. Relevance for Nutritional Strategies and Transition Toward Cocoa-Based Matrices

The strongest rationale for emphasizing oleic-rich lipid matrices in metabolic nutrition emerges from dietary intervention studies comparing monounsaturated fat-rich diets with higher-carbohydrate or higher-saturated fat dietary patterns. Among the foundational studies in this area is the KANWU trial, in which Vessby and colleagues showed that replacing saturated fat with monounsaturated fat improved insulin sensitivity in healthy subjects, particularly when total fat intake remained within a moderate range [70]. This study remains highly relevant because it demonstrated that the quality of dietary fat, not simply the amount, can alter insulin action in humans. It also provided one of the earliest controlled demonstrations that MUFA-rich diets may preserve insulin sensitivity better than saturated fat-rich diets when energy intake is stable [70].

Subsequent work extended this concept to populations with insulin resistance. Paniagua et al. studied insulin-resistant subjects and reported that a Mediterranean-style diet rich in monounsaturated fat improved insulin sensitivity and reduced postprandial glucose excursions compared with a high-carbohydrate diet [71]. In a related study, Paniagua et al. reported that an isocaloric monounsaturated fat-rich Mediterranean diet prevented central fat redistribution and the postprandial decrease in peripheral adiponectin expression and insulin sensitivity induced by a carbohydrate-rich diet in insulin-resistant subjects, suggesting that monounsaturated fat may modulate insulin action not only through acute substrate handling but also through longer-term changes in adipose tissue biology and metabolic partitioning [72]. These findings show that lipid quality can modify glycemic physiology at the level of both postprandial metabolism and whole-body insulin sensitivity.

Mechanistically, one of the most discussed explanations for these effects is the influence of oleic acid on membrane composition and cellular signaling. López et al. reviewed evidence indicating that dietary fatty acid composition can alter membrane phospholipid profiles, receptor microenvironment, and the function of proteins involved in insulin signaling [73]. Oleic acid may favor a membrane environment that is more compatible with insulin receptor activation and downstream signaling than saturated fatty acids, while also limiting the accumulation of lipotoxic intermediates such as ceramides and diacylglycerols that interfere with insulin action [73,75]. Although much of this mechanistic work derives from experimental models, it provides biological plausibility for the human intervention data and helps explain why oleic-rich matrices may be metabolically advantageous beyond their caloric contribution.

This mechanistic interpretation is reinforced by studies of postprandial lipemia. Pérez-Martínez et al. showed that the type of dietary fat consumed acutely influences postprandial insulin sensitivity, endothelial function, and inflammatory responses, with monounsaturated fat-rich meals generally producing a more favorable postprandial profile than saturated fat-rich meals in subjects with metabolic syndrome [74]. This is particularly relevant for a review centered on cocoa-based matrices, since these products are typically consumed in the postprandial context rather than as fasting interventions. If an oleic-rich lipid fraction can improve the acute vascular–metabolic response to a meal, then lipid reformulation becomes a central element of the matrix strategy rather than a secondary compositional detail.

Evidence from randomized trials and prospective analyses indicates that diets rich in monounsaturated fatty acids are associated with improvements in cardiometabolic risk factors, including beneficial effects on fasting insulin and insulin sensitivity indices in selected populations [75]. Schwingshackl et al. later showed that higher olive oil intake was associated with lower risk of type 2 diabetes in prospective analyses and intervention-based evidence, especially as part of Mediterranean dietary patterns [76]. Although these findings reflect complex dietary patterns rather than isolated fatty acids, they position oleic-rich lipid matrices within a clinically relevant nutritional context [75,76].

At the level of food formulation, this evidence has direct implications for cocoa-based products. Conventional chocolate matrices contain substantial amounts of fat, but the metabolic effect of that lipid fraction depends on its composition and on how it interacts with sugars, fibers, and polyphenols. Cocoa butter itself is unusual in that it contains a relatively high proportion of stearic acid together with oleic acid, and stearic acid is often considered less hypercholesterolemic than other saturated fatty acids [106]. However, when cocoa-based products are reformulated using oleic-rich lipids (such as high-oleic nut pastes, olive-derived lipid systems, or oleic-enriched fillings), the resulting matrix may differ substantially in its postprandial and long-term metabolic behavior. Oleic-rich lipid design should therefore be understood not as a simple substitution of fat sources, but as a strategy to modulate the interactions between lipid quality, glycemic response, satiety, and insulin sensitivity within the food matrix.

This point becomes even more important when cocoa products are intended for individuals with impaired glucose regulation. A cocoa matrix rich in flavanols but combined with a high glycemic load and an unfavorable lipid profile may fail to deliver the expected metabolic benefit. By contrast, a matrix in which polyphenols are combined with low-glycemic sweeteners, fermentable fibers, and oleic-rich lipids may provide a more coherent metabolic profile, attenuating postprandial glucose exposure while simultaneously favoring vascular and insulin-related pathways. Thus, the role of oleic-rich lipids in the present review is not to displace the importance of cocoa polyphenols, but to show how lipid architecture can either reinforce or dilute their physiological relevance.

### 8.2. Human Evidence: Promise, Inconsistency, and Context Dependence

Although the mechanistic and dietary rationale for oleic-rich lipid matrices is strong, the human evidence remains context-dependent and should not be interpreted as universally conclusive. One reason for this is that the effects of oleic acid are rarely studied in isolation. In practice, oleic-rich matrices are embedded within broader dietary patterns, and their physiological impact depends on what they replace (saturated fat, refined carbohydrate, or mixed energy intake) as well as on baseline insulin resistance, total energy balance, and intervention duration [70,75,76]. This means that improvements attributed to MUFA may result not only from the intrinsic biological properties of oleic acid, but also from the displacement of more deleterious macronutrient profiles.

This complexity is evident in the CORDIOPREV study and related Mediterranean diet research. In long-term dietary interventions, Mediterranean patterns rich in olive oil have been associated with improved insulin sensitivity, lower incidence of T2D, and favorable changes in metabolic syndrome traits [76,107]. Yet these benefits reflect the combined action of multiple dietary components, including polyphenols, fiber, lower glycemic load, and improved meal structure, not oleic acid alone [76,107]. However, this does not weaken the argument. On the contrary, it reinforces the matrix-based principle that oleic-rich lipids are metabolically relevant precisely because they operate within complex dietary systems rather than as isolated molecules.

Randomized feeding trials further highlight variability in response to oleic-rich diets. In some studies, oleic-rich diets improve fasting insulin sensitivity and postprandial metabolism; in others, the effects are modest or depend on subgroup characteristics. Vessby et al. showed that replacing saturated fat with monounsaturated fat improved insulin sensitivity, although this effect was not universal and disappeared when total fat intake was high. In addition, subsequent evidence from the LIPGENE study indicated that the metabolic phenotype of participants influences the response to dietary fat modification, suggesting that the benefits of increasing monounsaturated fat intake are more evident in specific insulin-resistant subgroups rather than being homogeneous across all individuals [108]. Similarly, López-Miranda and colleagues highlighted that postprandial responses to dietary fatty acid quality show substantial interindividual variability and are influenced by physiological, genetic, and pathological factors, including adiposity and baseline cardiometabolic status [109]. Collectively, these findings indicate that oleic acid should not be interpreted as a universally insulin-sensitizing nutrient, but as a context-dependent component whose metabolic effects are shaped by dietary composition, metabolic phenotype, and overall matrix structure.

Another relevant issue concerns the interaction between lipids and carbohydrate metabolism within mixed meals. Oleic-rich fats may improve postprandial glucose handling in part by slowing gastric emptying and altering the delivery of nutrients to the absorptive surface, but these effects are inseparable from the broader meal composition [74,110]. In cocoa-based products, where sugar, fiber, and particle structure all affect digestion, the impact of oleic-rich lipids cannot be assumed to replicate that observed in liquid test meals or Mediterranean diet interventions. A high-oleic cocoa spread, a nut-enriched dark chocolate, and a cocoa-based beverage stabilized with an oleic-rich emulsion are all “oleic-rich matrices”, but they are unlikely to behave identically from a glycemic standpoint. The final response depends on the interaction between lipid profile, carbohydrate displacement, food structure, and the presence of other bioactive compounds.

Insulin sensitivity represents a multifactorial outcome influenced by several overlapping mechanisms, including acute postprandial responses, long-term changes in ectopic fat accumulation, modulation of inflammatory tone, and interactions with other dietary components. For instance, olive oil-rich Mediterranean diets often improve insulin sensitivity while also reducing inflammation and improving endothelial function [76,107,111]. In cocoa-based matrices, similar synergies may occur if oleic-rich lipids are combined with flavanols, which have already been discussed in relation to nitric oxide signaling and vascular responses, and with fibers that slow carbohydrate delivery. This reinforces the idea that oleic-rich lipids should not be interpreted narrowly as “fat quality variables”, but as contributors to a broader matrix design that may influence insulin sensitivity at multiple levels.

Overall, current evidence supports a context-dependent interpretation. Oleic-rich lipid matrices appear metabolically favorable primarily when they replace saturated fats or high-glycemic carbohydrate profiles and when they are embedded within dietary patterns or formulations that support insulin sensitivity and vascular health [70,71,72,73,74,75,76,106,107,108,109,110,111]. Human intervention studies provide reasonably strong support for improved insulin-related and cardiometabolic outcomes in selected populations, especially within Mediterranean-style contexts [71,74,75,76,107,108]. However, the effects are not uniform, and the specific contribution of oleic acid is often difficult to separate from that of the food matrix and overall diet. This context dependence is not a limitation unique to oleic acid; rather, it is a defining feature of nutritional physiology and one of the reasons why a matrix-oriented approach is necessary.

Oleic-rich lipid matrices may contribute to insulin sensitivity not through a single dominant mechanism, but by improving the lipid quality of the formulation, reducing postprandial metabolic stress, supporting membrane and signaling physiology, and interacting favorably with other components of cocoa-based foods. Their value lies in helping to construct a matrix that is metabolically coherent, especially when paired with cocoa polyphenols, low-glycemic sweeteners, and appropriate fiber components. This formulation logic naturally leads to the next section, in which micronutrients and redox balance will be examined as additional determinants of metabolic regulation within cocoa-based plant matrices.

## 9. Micronutrients and Redox Balance in Metabolic Regulation

Micronutrients occupy a distinctive position within the metabolic architecture of cocoa-based plant matrices because their physiological relevance is not primarily determined by caloric contribution, but by their role as enzyme cofactors, modulators of redox homeostasis, regulators of insulin signaling, and determinants of mitochondrial and vascular function. In the context of the present review, their importance lies in the fact that oxidative stress and dysglycemia are not driven exclusively by excess substrate load or inflammatory activation, but also by the efficiency of endogenous antioxidant systems and the micronutrient-dependent pathways that sustain them. This is especially relevant for cocoa-derived foods, since cocoa naturally contains minerals such as magnesium, copper, iron, and smaller amounts of zinc, while reformulated plant matrices may also incorporate additional micronutrient-rich ingredients [25,26,27,112,113].

From a mechanistic perspective, micronutrients can influence metabolic regulation through at least three broad routes. First, they serve as structural or catalytic cofactors for antioxidant enzymes involved in the control of reactive oxygen species, such as glutathione peroxidases, superoxide dismutase, and other redox-sensitive systems. Second, they participate in insulin secretion, insulin receptor signaling, glucose transport, and intracellular phosphorylation events relevant to carbohydrate metabolism. Third, they may shape inflammatory tone, mitochondrial performance, and endothelial responsiveness, thereby influencing the broader metabolic milieu in which insulin resistance and glycemic dysregulation develop [112,113]. This distinction is important because micronutrients should not be conceptualized merely as “supportive nutrients” but as biologically active determinants of whether redox stress is amplified or buffered under conditions of metabolic overload.

Among the micronutrients most consistently linked to glucose metabolism, magnesium has attracted particular attention. Magnesium is required for ATP-dependent enzymatic reactions and plays a central role in insulin receptor phosphorylation, post-receptor signaling, membrane transport, and cellular glucose utilization [112,113]. Barbagallo and Dominguez emphasized that magnesium deficiency is common in individuals with type 2 diabetes and may aggravate insulin resistance, impaired insulin secretion, low-grade inflammation, and oxidative stress [112]. This relationship appears bidirectional, as insulin influences magnesium homeostasis while intracellular magnesium availability modulates insulin action [112,113].

Epidemiological evidence supports this association. In a meta-analysis of prospective cohort studies, Dong et al. reported that higher magnesium intake was associated with a lower risk of type 2 diabetes, suggesting that habitual magnesium consumption may have long-term metabolic relevance [114]. Although such observational findings cannot establish causality, they are consistent with the mechanistic literature and justify the interest in magnesium-containing matrices as part of preventive nutritional strategies [112,113,114]. Interventional evidence also points in the same direction. Simental-Mendía et al., in a systematic review and meta-analysis of randomized trials, concluded that magnesium supplementation improved insulin sensitivity and glycemic control, particularly in individuals with magnesium deficiency, insulin resistance, or type 2 diabetes [115]. Likewise, Mooren et al. showed that oral magnesium supplementation improved insulin sensitivity in non-diabetic but insulin-resistant individuals, reinforcing the view that magnesium may be particularly relevant in early metabolic dysfunction rather than only in overt diabetes [116].

This pattern is important for the present review because it illustrates a broader principle that applies to micronutrients in general: benefits are often more evident in those with existing deficiency, suboptimal status, or metabolic vulnerability. In other words, micronutrients do not act as universal pharmacological enhancers of metabolism, but as context-dependent regulators whose effects emerge more clearly when physiological reserves are impaired. For cocoa-based matrices, this means that the intrinsic mineral profile of the product may contribute to metabolic value, but the magnitude of the effect will depend on the baseline nutritional status and metabolic phenotype of the consumer.

Zinc represents another key micronutrient in metabolic regulation. Its relevance derives from its structural role in insulin storage and crystallization within pancreatic β-cells, its involvement in antioxidant defense, and its participation in signaling pathways linked to inflammation and glucose homeostasis [117,118]. Chausmer described zinc as a nutrient closely linked to insulin physiology, emphasizing its role in synthesis, secretion, and action [117]. Ranasinghe et al. reported that zinc plays an important role in insulin action, β-cell function, glucose homeostasis, and the pathogenesis of diabetes and its complications, supporting its relevance at the interface between redox regulation and glucose metabolism [118].

Human evidence on zinc remains suggestive but not fully consistent. In a systematic review and meta-analysis, Jayawardena et al. found that zinc supplementation improved glycemic control and promoted healthier lipid parameters in individuals with diabetes, although further studies were needed to clarify the exact biological mechanisms underlying these effects [119]. Rather than acting as an isolated intervention, the relevance of zinc lies in its role within the broader redox-metabolic environment, where micronutrient sufficiency conditions the physiological response to other matrix components, including polyphenols, fibers, and lipid composition.

Selenium introduces a more complex and more controversial dimension to this section. Its biological importance is well established: selenium is an essential constituent of selenoproteins, including glutathione peroxidases and thioredoxin reductases, which are major components of the cellular antioxidant machinery [120,121]. In principle, this would suggest that adequate selenium status should support protection against oxidative stress-related metabolic dysfunction. However, human literature has shown that the relationship between selenium and metabolic health is not linear. Rayman reviewed the broad role of selenium in human health and emphasized that both deficiency and excess may be biologically problematic, particularly because selenium operates within a narrow physiological range [120].

This issue became especially salient after the report by Stranges et al., who found in a randomized trial that long-term selenium supplementation was associated with a higher incidence of type 2 diabetes [122]. This finding has been widely debated, but it is highly relevant for the present review because it illustrates the danger of assuming that strengthening antioxidant defense through micronutrient supplementation will necessarily improve metabolic outcomes. Steinbrenner later explored this paradox mechanistically, arguing that selenium and selenoproteins may interfere with insulin-regulated carbohydrate and lipid metabolism in complex ways, including effects on redox signaling that are not uniformly beneficial when selenium exposure exceeds physiological requirements [121]. Thus, selenium serves as an important reminder that redox balance is not equivalent to indiscriminate antioxidant enhancement; metabolic regulation requires homeostasis, not maximal antioxidant input.

Chromium has also been investigated extensively in relation to glucose regulation, particularly because of its proposed role in potentiating insulin action. Although it is not typically classified as a redox-active micronutrient, chromium is often considered within metabolic nutrition because of its potential effects on insulin signaling and glucose disposal. Balk et al., in a systematic review of randomized trials, found inconsistent evidence for chromium supplementation on glucose metabolism and lipid outcomes, with some studies reporting benefit and others showing minimal or no effect [123]. Suksomboon et al. reached similarly cautious conclusions in a later meta-analysis, observing modest improvements in some glycemic variables but substantial heterogeneity and uncertain clinical significance overall [124]. This pattern illustrates a broader challenge in micronutrient research: physiological plausibility does not necessarily translate into consistent clinical efficacy, particularly when baseline nutritional status, dosage, and population characteristics vary across studies.

### 9.1. Relevance for Nutritional Strategies and Transition Toward Cocoa-Based Matrices

The nutritional relevance of these micronutrients becomes clearer when they are interpreted not as isolated supplements but as integral components of food matrices. Cocoa naturally contains appreciable amounts of magnesium and copper and, depending on origin and processing, can also contribute iron and other trace minerals [25,26,27,33,34]. This does not mean that cocoa should be presented as a micronutrient supplement in disguise. Rather, it means that the mineral composition of cocoa-based matrices may participate in the broader metabolic context in which polyphenols, fibers, sweeteners, and lipids act. A cocoa-based food rich in flavanols but poor in micronutrient quality may not exert the same integrated physiological effect as a matrix in which redox-active minerals, low-glycemic carbohydrates, and favorable lipids are simultaneously optimized.

Copper deserves mention in this regard because of its role in redox biology. UriuAdams and Keen reviewed the relationship between copper, oxidative stress, and human health, emphasizing that copper is essential for antioxidant defense through enzymes such as Cu/Zn-superoxide dismutase, but that copper imbalance can also contribute to oxidative injury [125]. This duality mirrors the broader theme of the section: micronutrients are beneficial when they support physiological redox control, but potentially deleterious when imbalanced. In cocoa-based matrices, the relevance of copper is therefore not simply quantitative, but functional, depending on whether the matrix contributes to balanced micronutrient exposure within an overall diet.

Iron introduces another important layer of complexity. While essential for oxygen transport, mitochondrial function, and enzymatic activity, excess iron can promote oxidative stress through Fenton chemistry and has been associated with insulin resistance and metabolic dysfunction [54]. This highlights that micronutrient effects are not uniformly beneficial but depend on dose, bioavailability, host status, and the balance between physiological requirement and redox burden.

From a formulation perspective, these observations have practical implications. If cocoa-based foods are being designed for metabolic health, the micronutrient profile of the matrix should be considered part of the functional architecture rather than an incidental compositional feature. A matrix that provides meaningful magnesium and zinc, avoids excessive glycemic burden, preserves flavanols, and incorporates appropriate fiber and lipid components may offer a more coherent metabolic profile than one focused on a single “hero ingredient.” This is particularly important because many redox-related pathways are enzyme-dependent and therefore constrained by cofactor availability. In this sense, micronutrients may act as permissive factors that enable or sustain the beneficial effects of other bioactive compounds already discussed in the manuscript.

### 9.2. Micronutrients in Human Evidence: Context Dependence and Homeostatic Limitations

The human evidence on micronutrients is characterized by both promise and inconsistency. Magnesium has the strongest and most coherent signal, with observational studies, supplementation trials, and mechanistic work broadly pointing in the same direction [112,113,114,115,116]. Even here, however, the most favorable effects are usually observed in people with low magnesium status, insulin resistance, or diabetes, rather than across all populations indiscriminately [115,116]. Zinc also shows potentially beneficial effects, but the evidence is less consistent and often limited by trial quality and baseline nutritional heterogeneity [118,119]. Selenium remains particularly controversial because its antioxidant role coexists with evidence that excess exposure may increase diabetes risk [120,121,122]. Chromium continues to occupy a marginal and uncertain position because clinically meaningful benefits remain difficult to establish consistently across trials [123,124].

These do not indicate that micronutrients are irrelevant, but rather that their effects are constrained by physiological homeostasis and contextual factors. Their metabolic impact depends on baseline nutritional status, disease phenotype, dietary background, and mode of delivery. The physiological meaning of a micronutrient may differ substantially when it is consumed as part of a complex food, where it interacts with polyphenols, fibers, fats, and digestion kinetics, compared with when it is delivered as a high-dose isolated supplement. In cocoa-based foods, the relevant question is therefore not whether magnesium, zinc, selenium, or chromium “treat” insulin resistance in isolation, but whether their presence within the matrix contributes to a more favorable redox-metabolic environment.

Taken together, current evidence indicates that micronutrients are best understood as modulators of redox balance and metabolic competence rather than as stand-alone antidiabetic agents, as shown in Table 5 [54,112,113,114,115,116,117,118,119,120,121,122,123,124,125]. Magnesium appears most consistently relevant to insulin sensitivity and glucose control, zinc may support β-cell and antioxidant function, selenium illustrates the importance of physiological balance in redox regulation, and chromium remains mechanistically interesting but clinically uncertain [112,113,114,115,116,117,118,119,120,121,122,123,124]. Within cocoa-based plant matrices, these micronutrients may enhance metabolic quality when they contribute to endogenous antioxidant defense, insulin signaling efficiency, and vascular-metabolic homeostasis. This interpretation also preserves coherence across the manuscript: whereas previous sections focused on carbohydrate kinetics, microbial mediation, and lipid architecture, the present section highlights the cofactor-dependent biochemical infrastructure that supports redox and metabolic regulation. This sets the stage for the next section, in which the manuscript will move from individual matrix components toward multi-component synergies and the integrated behavior of cocoa-based functional systems.

## 10. Multi-Component Synergies in Plant-Based Functional Matrices

Cocoa-based plant matrices contain several functional components with potential metabolic relevance, including cocoa polyphenols, non-glycemic sweeteners, prebiotic fibers, viscous soluble fibers, oleic-rich lipids, and micronutrients involved in redox regulation. Although these compounds have been widely studied individually, their physiological effects cannot be fully understood when examined in isolation. Nutritional science increasingly recognizes that the biological activity of food-derived compounds depends strongly on the structural context in which they are delivered. Within a structured food matrix, interactions among components influence digestion, bioaccessibility, postprandial metabolic kinetics, gut microbial metabolism, intracellular signaling pathways, and tissue-level metabolic responses [21,22,23,24]. As a result, the metabolic effects of cocoa-based systems may arise from additive, complementary, or synergistic interactions between matrix constituents rather than from the action of a single compound alone.

This perspective aligns with a broader shift from reductionist to matrix-oriented nutrition, in which the health effects of foods are understood as the result of coordinated interactions among multiple components. The food matrix is therefore not a passive carrier but an active determinant of nutrient release, accessibility, and physiological response [21,22,23]. Within this framework, the metabolic performance of cocoa-based formulations should be interpreted as a systems property of the matrix rather than the sum of isolated ingredients.

This issue is particularly relevant in metabolic disease. Hyperglycemia, oxidative stress, endothelial dysfunction, inflammation, altered gut-derived signaling, and insulin resistance do not arise from a single defective pathway and are therefore unlikely to be optimally modulated by compounds acting through only one route [9,10,11,12,13,14,15,16,17,18]. Plant-based matrices may offer an advantage precisely because they can target several dimensions of metabolic dysfunction simultaneously. A formulation that lowers glycemic load, preserves polyphenol bioactivity, provides fermentable substrates, improves lipid quality, and contributes redox-relevant micronutrients may influence postprandial physiology, endothelial function, inflammatory tone, and longer-term insulin sensitivity in a more coherent manner than any isolated intervention. The challenge, therefore, is not simply to identify “beneficial ingredients”, but to understand how these ingredients interact within the matrix and whether those interactions improve or impair metabolic function.

### 10.1. Synergy as a Matrix-Dependent Metabolic Property

At a mechanistic level, synergy in plant-based functional matrices may arise through several distinct but overlapping routes. One is a complementary pathway targeting, whereby different matrix components act on different stages of the same physiological process. In a cocoa-based formulation, non-glycemic sweeteners may reduce the immediate glycemic stimulus of the product, viscous soluble fibers may slow gastric emptying and glucose absorption, cocoa flavanols may improve endothelial nitric oxide signaling and postprandial vascular responsiveness, and fermentable substrates may subsequently influence microbial fermentation, gut-derived metabolites, and inflammatory tone in the distal intestine [14,15,16,31,37,53,60,61,62,63,64,65,66,67,68,69,89,90,91,92,93,94,95,96,97,98,99,100,101,102,103,104,105]. Although these actions are mechanistically distinct, their combined effects can produce a more favorable postprandial metabolic profile than any individual component alone.

A second route is bioaccessibility-dependent interaction, in which one component alters the release, stability, or intestinal availability of another. This principle is central to food matrix science. Parada and Aguilera emphasized that food microstructure affects nutrient bioavailability by determining how nutrients and phytochemicals are released during digestion and made accessible to intestinal absorption [55]. In cocoa-based systems, lipid phase organization, particle size, hydration, emulsification, and fiber-associated viscosity can all modify the extent to which polyphenols, minerals, and carbohydrate fractions become available at different stages of digestion. Thus, the metabolic effect of cocoa flavanols cannot be fully separated from the matrix conditions that govern their accessibility and their interaction with other macronutrients.

A third route is kinetic synergy, whereby components modify the temporal pattern of metabolic exposure rather than acting on the same molecular target. This is especially relevant for glycemic regulation. A matrix containing low-glycemic sweeteners, β-glucans or other viscous fibers, and a favorable lipid phase may not dramatically alter fasting biomarkers after a single exposure, yet it may substantially reshape the kinetics of postprandial glucose appearance, insulin secretion, gastric emptying, and vascular stress. Such changes are metabolically important because, as discussed earlier, glycemic variability itself contributes to oxidative and endothelial injury [14,15,16]. In this sense, synergy may be expressed not only in the absolute magnitude of an effect, but also in the redistribution of metabolic stress over time.

A fourth route involves convergence at the level of host–microbiota signaling. Cocoa polyphenols, fermentable fibers, and certain low-digestible carbohydrate fractions may all reach the colon and undergo microbial transformation, thereby influencing bacterial composition and metabolite production [32,89,97]. Tzounis et al. showed that high-cocoa-flavanol intake increased fecal bifidobacteria and lactobacilli while reducing clostridia in healthy humans, together with favorable changes in circulating triglycerides and C-reactive protein [89]. Sorrenti et al. later reviewed the bidirectional relationship between cocoa polyphenols and gut microbiota, highlighting that cocoa constituents may simultaneously modulate microbial composition and undergo microbial biotransformation into smaller metabolites with distinct biological activity [97]. When such effects are combined with established prebiotic substrates, such as inulin-type fructans, the resulting matrix may generate a more complex and potentially more durable modulation of gut–host metabolic signaling than either component alone.

This systems-oriented interpretation is also supported by the broader “whole food” concept. Williamson argued that the physiological actions of polyphenols depend not only on their intrinsic chemistry but also on transport, metabolism, tissue exposure, and interactions with co-ingested nutrients [39]. Similarly, the metabolic effects of β-glucans depend strongly on viscosity and matrix structure rather than nominal fiber dose alone [63,64,65,66,67,68,69,99,100,101,102,103,104,105]. Oleic-rich lipids appear to improve insulin sensitivity more consistently when embedded within mixed matrices or dietary patterns that displace less favorable fats or refined carbohydrates [70,71,72,73,74,75,76,106,107,108,109,110,111]. Micronutrients, in turn, often exert context-dependent effects that become clearer when nutritional status is suboptimal or when cofactor-dependent redox systems are under stress [54,112,113,114,115,116,117,118,119,120,121,122,123,124,125]. These examples converge on the same principle: the metabolic significance of a bioactive compound is inseparable from the matrix in which it is delivered.

These considerations also have direct implications for formulation logic. A metabolically coherent cocoa-based matrix may combine cocoa flavanols as redox- and endothelial-active compounds; a non-glycemic sweetener to reduce acute glycemic burden; fermentable fibers to support selective microbial utilization; β-glucans or other viscous soluble fibers to attenuate nutrient absorption kinetics; an oleic-rich lipid phase to improve fat quality; and selected micronutrients to support redox and insulin-related pathways [36,37,38,39,40,41,42,43,44,45,46,47,48,49,50,54,60,61,62,63,64,65,66,67,68,69,70,71,72,73,74,75,76,77,78,79,80,81,82,83,84,85,91,92,93,94,95,96,99,100,101,102,103,104,105,106,107,108,109,110,111,112,113,114,115,116,117,118,119,120,121,122,123,124,125]. Such a design does not imply that all effects will necessarily be synergistic in the strict pharmacological sense. It does, however, support the more realistic nutritional claim that multiple components can be organized so that they act on complementary physiological targets within the same matrix. This type of coordinated formulation is more aligned with real-world food design than the isolated compound paradigm that still dominates much of the intervention literature.

### 10.2. Human Evidence on Multi-Component Synergy: Translational Relevance and Constraints

Although the theoretical basis for synergy in plant-based matrices is strong, direct human evidence specifically demonstrating synergistic interactions remains more limited than the mechanistic plausibility might suggest. One reason is methodology: most clinical trials are designed to test single ingredients, single supplements, or relatively simple substitutions rather than integrated multi-component matrices. As a result, much of the evidence for synergy in this field is inferential rather than experimentally isolated. Nevertheless, several lines of research support the translational relevance of matrix-level thinking.

Mediterranean dietary studies provide an important parallel. The protective effects of Mediterranean patterns cannot reasonably be attributed to a single nutrient, but rather to the combined action of polyphenol-rich plant foods, oleic-rich lipids, fermentable fibers, lower glycemic load, and micronutrient density [19,20,75,76]. In metabolic terms, this offers a useful analog for cocoa-based matrices: health effects may emerge from coordinated compositional design rather than from a single “active principle”. Although cocoa-based formulations are more product-specific than whole dietary patterns, the same principle applies. A cocoa matrix that combines preserved flavanols with reduced glycemic load, added fermentable or viscous fibers, a favorable lipid profile, and supportive micronutrient composition may plausibly reproduce, on a smaller scale, the systems logic that underlies healthier dietary structures.

Human studies on cocoa also suggest strongly matrix-dependent behavior. Grassi and colleagues showed that dark chocolate rich in polyphenols improved insulin sensitivity and vascular function more than white chocolate, indicating that not all chocolate matrices are metabolically equivalent [36,42]. Muniyappa et al. demonstrated that cocoa improved insulin-mediated vasodilation without clearly improving whole-body insulin resistance, suggesting that matrix effects may emerge first in vascular domains rather than in conventional glycemic endpoints [43]. Tzounis et al. provided evidence that cocoa flavanols can modify the gut microbiota [89], while other studies have shown that the metabolic relevance of cocoa depends heavily on processing, sugar content, and formulation [37,38,40,50]. Collectively, these findings support a critical point for the present review: even when the same botanical ingredient is used, the final physiological effect depends on the surrounding matrix.

This interpretation is particularly relevant for functional food design. A cocoa-based product formulated with sucrose and limited structural fiber may generate a substantially different metabolic response from a cocoa-based product formulated with erythritol or stevia, enriched with fermentable and viscous fibers, and embedded in an oleic-rich lipid phase. The former may deliver flavanols yet still impose a considerable postprandial glycemic burden; the latter may better align with the metabolic objectives of antioxidant and antidiabetic formulation by simultaneously lowering glycemic load, slowing nutrient absorption, supporting endothelial responsiveness, and potentially enhancing microbiota- and redox-related pathways. In this sense, synergy is not an abstract biochemical label; it is a practical design principle.

At the same time, the formulation perspective introduces important constraints. Not all theoretically favorable combinations produce acceptable foods. Viscous fibers may impair texture, high-intensity sweeteners may alter aftertaste, polyphenols may contribute bitterness or astringency, and lipid restructuring can affect mouthfeel, stability, and shelf life. This is one reason why translational nutrition must remain connected to food science. As Aguilera emphasized, the matrix determines not only nutrient delivery but also processability and sensory behavior [23]. Therefore, the challenge in cocoa-based functional foods is not simply to maximize the concentration of beneficial compounds, but to achieve a matrix architecture that is physiologically coherent, technologically feasible, and sensorially acceptable.

Another major issue is the difficulty of distinguishing additive from truly synergistic effects in human studies. If a cocoa-based product with low-glycemic sweeteners and soluble fibers improves postprandial glucose compared with conventional chocolate, it may be difficult to determine whether the benefit arises because the sweetener lowered sugar exposure, because the fiber increased viscosity, because the lipid profile altered nutrient delivery, or because the combination created an interaction that exceeded the sum of its parts. This problem is common in functional food research and does not invalidate the matrix concept. However, it does mean that claims of synergy must be made carefully and grounded in mechanistic plausibility, comparative formulation studies, and physiological coherence rather than rhetorical appeal.

From the perspective of metabolic regulation, the strongest translational argument for multi-component synergy is that metabolic disease itself is multifactorial. Insulin resistance develops in a physiological environment shaped by glycemic variability, lipid overload, inflammation, endothelial dysfunction, microbiota-derived signaling, and redox imbalance [9,10,11,12,13,14,15,16,17,18]. It is therefore reasonable to hypothesize that a matrix acting on several of these processes simultaneously may have greater practical relevance than one acting on only one. This is particularly true for foods intended for habitual consumption, where modest repeated effects on digestion, glycemia, vascular function, inflammation, and microbial metabolism may accumulate over time. A cocoa-based matrix that repeatedly lowers post-prandial glycemic excursion, preserves endothelial function, improves lipid quality, and supports microbial fermentation may be more meaningful in the long term than a product that delivers high flavanol content but leaves the rest of the metabolic profile unchanged.

Taken all together, the available evidence supports the view that multi-component synergy in plant-based functional matrices is biologically plausible, nutritionally relevant, and central to the design logic of cocoa-based products [21,22,23,24,31,37,39,53,54,55,60,61,62,63,64,65,66,67,68,69,70,71,72,73,74,75,76,89,90,91,92,93,94,95,96,97,98,99,100,101,102,103,104,105,106,107,108,109,110,111,112,113,114,115,116,117,118,119,120,121,122,123,124,125]. However, this concept should be interpreted carefully: not as a vague assumption that the simple combination of several “healthy” ingredients is always beneficial, but as the coordinated interaction of components whose joint effects on digestion, signaling, microbial metabolism, and postprandial physiology may be more coherent than expected from isolated compounds alone. Within the architecture of this review, this section provides the conceptual bridge from individual matrix components toward real-world functional food design. The next section will build on that bridge by addressing research gaps and future directions for cocoa-based matrices for anti-diabetic nutritional strategies, where issues of target population, feasibility, positioning, and clinical application become even more explicit.

## 11. Research Gaps and Future Directions

The literature reviewed throughout this manuscript supports the biological plausibility of cocoa-based plant matrices as modulators of oxidative stress, postprandial glycemia, endothelial function, microbial signaling, and selected markers of insulin sensitivity. At the same time, the field remains characterized by substantial heterogeneity in formulation, processing, dose, study design, and clinical endpoints. As a result, one of the principal research gaps is not the absence of mechanistic hypotheses, but the insufficient integration of these hypotheses into standardized, matrix-specific, and clinically relevant experimental models. In other words, the field does not primarily need more isolated claims that cocoa polyphenols, fibers, or lipid components may be beneficial; it needs better-designed studies capable of determining under which compositional and technological conditions a cocoa-based matrix becomes metabolically meaningful in humans.

A major gap concerns the characterization of the cocoa fraction itself. One of the recurring limitations in the literature is that studies often refer to “cocoa”, “dark chocolate”, or “flavanol-rich products” without adequately standardizing or reporting the technological steps that determine flavanol retention and bioavailability. This issue is not trivial. Fermentation, drying, roasting, alkalization, and downstream industrial processing can substantially modify the concentration and profile of monomeric flavanols and procyanidins, thereby changing the biological potential of the final matrix [27,33,34,35]. Miller et al. showed that alkalization markedly reduces flavanol content and antioxidant capacity in commercial cocoa powders, illustrating how processing can weaken the physiological rationale of a cocoa-based intervention even before it reaches the consumer [56]. Payne et al. similarly demonstrated that fermentation, roasting, and Dutch processing alter catechin and epicatechin concentrations across cocoa ingredients, making it difficult to compare intervention studies that do not adequately report manufacturing history [51]. Kothe et al. further showed that roasting affects epimerization and degradation patterns of cocoa flavanols, reinforcing the view that processing severity is directly linked to the chemical identity of the bioactive fraction [52]. Thus, future research should move toward matrix characterization protocols that include not only nominal cocoa content, but also flavanol profile, processing history, degree of alkalization, and, where possible, food-structure descriptors relevant to digestion and bioaccessibility.

A second major gap concerns the predominance of single-ingredient or single-domain interventions over integrated matrix studies. Much of the current evidence has been generated using isolated flavanol extracts, purified fibers, stand-alone sweeteners, or relatively simple dietary substitutions. While such designs are useful for mechanistic clarification, they do not adequately reflect the physiological behavior of real cocoa-based functional foods. Future work should therefore prioritize intervention studies comparing whole matrices that differ in coordinated ways—for example, in sweetening systems, soluble fiber architecture, lipid phase composition, and flavanol preservation—rather than continuing to infer the expected effect of real foods from isolated-ingredient studies. This is especially important because the concept of synergy discussed in the previous section remains more often assumed than directly tested. Comparative trials designed around matrix-level contrasts would make it possible to determine whether combinations of low-glycemic sweeteners, fermentable fibers, viscous fibers, oleic-rich lipids, and preserved cocoa flavanols confer advantages beyond the sum of their parts.

A third gap concerns the selection of clinical endpoints. As discussed in earlier sections, many cocoa-based or matrix-based interventions may exert their earliest and most reproducible effects on postprandial glucose handling, endothelial function, inflammatory tone, or microbial metabolites rather than on fasting glucose or HbA1c alone [14,15,16,41,43,44,89,95]. Yet many nutritional trials continue to prioritize chronic glycemic indices without capturing the acute physiological mechanisms through which these foods are most likely to act. Future studies should therefore integrate more sensitive endpoint frameworks, including continuous glucose monitoring, postprandial insulin and incretin responses, endothelial function testing, lipidomic and metabolomic profiling, oxidative stress biomarkers, and microbiota-derived metabolites. Such an approach would be more aligned with the actual physiology of cocoa-based matrices and would reduce the risk of dismissing biologically relevant interventions simply because fasting endpoints remain unchanged over short periods.

A fourth gap lies in population stratification. Literature consistently suggests that the metabolic effects of cocoa flavanols, soluble fibers, prebiotics, oleic-rich lipids, and selected micronutrients are strongly phenotype-dependent. Individuals with prediabetes, insulin resistance, dyslipidemia, adiposity-associated inflammation, or micronutrient insufficiency may respond differently from metabolically healthy individuals [42,65,66,67,93,94,95,96,115,116]. This implies that future research should move beyond broadly heterogeneous cohorts and toward better phenotyped populations. The personalized nutrition literature already provides a useful conceptual framework for this transition. Zee-vi et al. showed that postprandial glycemic responses vary substantially between individuals and can be predicted using integrated phenotypic and microbiome-related variables [126]. Berry et al. later confirmed, in the PREDICT program, that postprandial glucose, lipid, and insulin responses show marked interindividual variability even under standardized meal conditions [127]. Ordovas et al. argued that such findings support a shift toward more personalized nutritional approaches grounded in phenotype, genotype, lifestyle, and microbiome context [128]. Within the field of cocoa-based matrices, this suggests that future antidiabetic strategies may benefit from stratifying participants by dysglycemic phenotype, postprandial response pattern, microbiota configuration, or baseline diet quality rather than assuming uniform physiological responsiveness.

A fifth research gap concerns the underdevelopment of sustainability-linked metabolic nutrition. Most studies on cocoa and metabolic health still evaluate efficacy in isolation from sourcing models, environmental impact, and food-system substitution. Yet from a translational perspective, future functional foods will likely be judged not only by their physiological effects, but also by their capacity to replace higher-impact ingredients with lower-footprint alternatives while maintaining nutritional quality. This introduces an important next-generation research question: can cocoa-based functional matrices be designed to combine metabolic benefit with lower environmental burden using ingredients derived from low-impact crops, legumes, carob, microalgae, or salt-tolerant coastal plants? Recent work on food-system sustainability suggests that this question is scientifically and strategically relevant. Clark et al. showed that foods differ markedly in their combined health and environmental impacts, reinforcing the need for integrative frameworks rather than nutrient-only evaluations [129]. Caporgno and Mathys reviewed the incorporation of microalgae into innovative foods and emphasized their promise as sustainable ingredients with functional and nutritional potential [130]. Ventura and Sagi highlighted the agronomic and ecological value of halophyte crops such as Salicornia and related salt-tolerant species, which may become relevant in future food systems facing freshwater and land constraints [131]. Within the present review, these lines of work suggest that a future direction for cocoa-based matrices is not merely compositional optimization, but the development of formulations that align metabolic functionality with regenerative or lower-impact ingredient sourcing. Such a direction would be especially relevant for products designed to replace conventional high-sugar confectionery or other metabolically unfavorable snack formats.

A sixth gap concerns the absence of robust translational frameworks for formulation optimization. Nutritional science has often progressed by testing one ingredient at a time, but the future of matrix-based interventions will likely require more integrative development models capable of combining metabolic, sensory, technological, and sustainability data. This is where digital and data-driven approaches may become increasingly important. Precision nutrition research already points toward the value of multidimensional prediction models for postprandial responses and dietary personalization [126,127,128]. The next methodological step may be the application of such logic to matrix design itself, using integrated datasets on glycemic impact, fiber functionality, flavanol stability, lipid composition, sensory performance, and environmental metrics to guide formulation decisions. In practice, this would mean moving from static formulation toward adaptive formulation science, where digital tools help identify ingredient combinations most likely to achieve targeted metabolic outcomes while preserving product feasibility. Although this field is still emerging, it may become especially relevant for cocoa-based systems, where small changes in sugar replacement, fiber type, or processing conditions can substantially alter both physiological and sensory behavior.

A seventh unresolved issue is the need for real-world implementation studies. Even the most mechanistically elegant cocoa-based formulation will have limited clinical significance if it is not accepted, tolerated, and habitually consumed by the target population. Future research should therefore incorporate pragmatic trials in settings that reflect real use conditions, including outpatient metabolic clinics, workplace nutrition programs, sports and wellness environments, hospital-adjacent dietary interventions, and preventive health settings. Such studies should examine not only metabolic markers, but also adherence, satiety, sensory satisfaction, product substitution patterns, and behavioral compensation. This is especially important for cocoa-based foods because they occupy a consumption space associated with pleasure and indulgence. Their translational promise lies precisely in the possibility of replacing metabolically unfavorable products with more coherent alternatives without requiring high levels of behavioral sacrifice.

Finally, there is a broader conceptual gap that affects the field as a whole: the tendency to frame matrix-based nutritional strategies either as overpromising “functional foods” or as underpowered dietary adjuncts. The literature reviewed here suggests that cocoa-based matrices should be positioned somewhere between these extremes. They are unlikely to function as stand-alone treatments for type 2 diabetes, yet they may still be physiologically relevant if they repeatedly reduce postprandial stress, improve dietary quality, and support vascular-metabolic resilience over time. Future directions should therefore focus less on attempting to convert cocoa-based foods into quasi-pharmaceutical agents and more on determining how such matrices can be integrated into realistic preventive and adjunctive nutritional strategies for dysglycemia.

Taken together, the most important future directions in this field can be summarized as follows: improved standardization of cocoa processing and flavanol preservation; more matrix-level comparative interventions; richer end-point selection emphasizing postprandial and systems biology markers; better phenotype-based stratification; integration of sustainability and ingredient-substitution logic into formulation science; use of digital and precision-nutrition approaches to optimize matrix architecture; and pragmatic implementation studies in real-world settings [51,52,56,126,127,128,129,130,131]. These priorities do not replace the mechanistic questions addressed earlier in the review; they extend them into the translational domain where cocoa-based plant matrices may become genuinely relevant as part of antidiabetic nutritional strategy. The final section will therefore synthesize the main conclusions emerging from the review and position cocoa-based functional matrices within the broader landscape of metabolic nutrition.

### 11.1. Chemical and Biological Horizons

The evidence reviewed throughout this manuscript identifies not only the potential metabolic relevance of cocoa-based plant matrices but also the substantial gaps that remain before such matrices can be considered established tools for antidiabetic nutritional strategies. Rather than summarizing what has already been discussed in the Research Gaps section, this Perspectives section aims to articulate the most promising directions from two complementary standpoints: the chemical characterization and optimization of cocoa-derived bioactives, and the clinical and biological research needed to translate matrix-based mechanistic insights into meaningful human outcomes.

### 11.2. Chemical Perspective

From a chemistry and food science standpoint, one of the most pressing priorities is the standardization and characterization of cocoa flavanol content across the chain from bean to finished product. As discussed in Section 10, processing steps including fermentation, drying, roasting, and alkalization substantially alter the flavanol profile and can reduce epicatechin and procyanidin concentrations by 60–90% compared to raw cacao [51,52,56]. Future research should develop and validate analytical protocols capable of quantifying not only total flavanol content but also the specific distribution of monomers, oligomers, and high-molecular-weight polymers—since these fractions differ in bioavailability, intestinal metabolism, and biological activity.

SAR analysis represents an additional chemical frontier that remains underdeveloped in the cocoa literature. Emerging data suggest that the position and number of hydroxyl groups on the flavanol skeleton, the degree of polymerization, and the stereochemistry at the C-2 and C-3 positions influence antioxidant capacity, protein binding, and receptor interactions. The 3’,4’-catechol moiety characteristic of (−)-epicatechin appears particularly important for metal chelation, radical scavenging, and eNOS activation. Procyanidin oligomers with increasing chain length show differential activity at AMPK, PI3K/Akt, and GLUT4-related pathways, though their lower bioavailability compared to monomers must be carefully considered when extrapolating in vitro SAR findings to in vivo conditions. Future chemical research should therefore aim to characterize not only native flavanol structures but also the profile and biological activity of gut-derived colonic metabolites—including phenyl-γ-valerolactones, phenylpropionic acid derivatives, and simple phenolic acids—which may represent the biologically active fraction in systemic tissues after oral consumption.

At the level of food matrix engineering, future chemical perspectives include the development of encapsulation and delivery systems capable of protecting flavanols from thermal and alkaline degradation during processing, improving their bioaccessibility in the gastrointestinal tract, and enabling targeted release in the small intestine or colon depending on the desired biological effect. The design of matrix architectures that preserve viscous fiber functionality, protect polyphenol bioactivity, ensure colonic delivery of prebiotic substrates, and incorporate oleic-rich lipid phases within a technologically feasible and sensorially acceptable format represents one of the most complex but scientifically rewarding challenges for functional food chemistry applied to metabolic health.

### 11.3. Biological and Clinical Perspective

From a biological and clinical research standpoint, the primary need is for standardized human intervention studies specifically designed to test complete cocoa-based matrices rather than individual extracted components. The overwhelming majority of existing evidence on flavanols, fibers, and non-glycemic sweeteners comes from supplementation studies using isolated or purified ingredients. While these provide mechanistic clarity, they do not adequately reflect the physiological behavior of real cocoa-based foods. Future studies should compare matrices that differ in a coordinated and pre-specified manner—for example, simultaneously varying the sweetening system (sucrose vs. erythritol + stevia), the fiber architecture (absent vs. fermentable vs. viscous), and the lipid profile (cocoa butter vs. oleic-enriched)—while maintaining a standardized flavanol content. Such designs would allow researchers to assess whether coordinated formulation strategies produce metabolic benefits that exceed those of conventional chocolate or isolated-ingredient interventions.

The selection of clinical endpoints is equally critical. Given that the most consistent effects of cocoa flavanols are observed on endothelial function, nitric oxide-related signaling, and postprandial oxidative stress rather than on conventional glycemic indices such as fasting glucose or glycated hemoglobin (HbA1c), future trials should integrate more sensitive and mechanistically relevant outcome frameworks. These include continuous glucose monitoring (CGM) for glycemic variability assessment, flow-mediated dilation and reactive hyperemia as endothelial function markers, lipidomic and metabolomic profiling of postprandial responses, quantification of plasma and urine oxidative stress biomarkers (8-isoprostane, 8-OHdG, FRAP, protein carbonyls), assessment of gut microbiota composition and function (16S rRNA sequencing; untargeted metabolomics), and homeostatic model assessment of insulin resistance (HOMA-IR) alongside C-peptide and incretin measurements. Such multidimensional endpoint frameworks would allow cocoa-based interventions to be evaluated across the full biological spectrum relevant to their proposed mechanisms, rather than being dismissed when fasting glucose fails to change over short periods.

Population stratification represents perhaps the most transformative clinical research direction for this field. Current evidence consistently suggests that the effects of flavanols, fibers, prebiotics, and MUFA-rich lipid matrices are more pronounced in individuals with pre-existing metabolic risk, including those with prediabetes, insulin resistance, obesity-associated inflammation, dyslipidemia, or microbiota dysbiosis. Future trials should therefore stratify participants by dysglycemic phenotype, baseline microbiome diversity and composition, postprandial response profiles (using CGM or mixed-meal tests), dietary quality indices, and micronutrient status. This personalized nutrition approach, informed by precision nutrition frameworks [126,127,128], would make it possible to identify the specific populations most likely to benefit from cocoa-based matrix interventions and to design products and dietary recommendations that are matched to individual metabolic contexts.

Finally, pragmatic implementation studies in real-world settings represent an underexplored but essential research direction. Even the most metabolically coherent cocoa-based formulation will have limited public health relevance if it fails to be habitually consumed, accepted, or substituted for less favorable dietary alternatives in real daily life. Studies conducted in preventive health settings, outpatient metabolic clinics, sports and wellness programs, and workplace nutrition initiatives are needed to evaluate not only metabolic biomarkers but also adherence, satiety, behavioral compensation, dietary substitution patterns, and quality of life outcomes. The translational promise of cocoa-based functional foods lies precisely in their capacity to replace metabolically unfavorable products while being perceived as pleasurable—a positioning that requires both scientific rigor and consumer-centered design.

## 12. Conclusions

Cocoa-based plants provide a coherent and mechanistically grounded framework for developing nutritional strategies targeting oxidative stress, postprandial glycemic burden, and endothelial dysfunction in metabolic disorders. The evidence reviewed demonstrates that their metabolic effects do not arise from a single bioactive component, but from matrix-dependent interactions among cocoa flavanols, non-glycemic sweeteners, functional fibers, oleic-rich lipids, and redox-active micronutrients.

Mechanistically, these matrices influence metabolic health by attenuating inflammatory signaling, preserving nitric oxide bioavailability, modulating nutrient absorption kinetics, and supporting microbiota-derived metabolic pathways. Although individual effects are often modest and context-dependent, their coordinated action can become metabolically meaningful, particularly in individuals with insulin resistance or early T2D.

However, the current evidence base highlights that the physiological efficacy of cocoa-based products is highly dependent on processing methods, formulation, and target population phenotypes. Future research must prioritize standardized matrix characterization, integrated multi-component clinical trials, and translationally relevant postprandial endpoints. Ultimately, while cocoa-based plant matrices are not stand-alone therapeutic agents, their rational formulation offers a valuable, synergistic tool within broader cardiometabolic and antidiabetic dietary strategies.

## Figures and Tables

**Table 5:** Micronutrient-dependent modulation of redox balance and glucose metabolism: mechanistic roles, human evidence, and contextual interpretation.

Micronutrient	Mechanistic Role (Redox & Metabolism)	Human Evidence (Key Findings)	Consistency of Evidence	Contextual Interpretation (Narrative Insight)	Key References
Magnesium	Cofactor in ATP-dependent enzymes; regulates insulin receptor phosphorylation; modulates GLUT activity; influences mitochondrial function and inflammatory tone	↓ Risk of T2D in cohort studies; improves insulin sensitivity and glycemic control in RCTs (especially deficient/IR subjects)	High consistency across epidemiology + RCTs	Acts as a rate-limiting cofactor in insulin signaling; effects are conditional on deficiency; strongest micronutrient signal in metabolic regulation	[112,113,114,115,116]
Zinc	Structural component of insulin storage (β-cells); antioxidant defense (SOD-related); modulates inflammatory signaling and insulin pathways	Modest improvements in fasting glucose, HbA1c and lipids; heterogeneous results across trials	Moderate consistency; variable effect sizes	Functions at β-cell–redox interface; effects depend on baseline status, dose, and metabolic phenotype; supportive but not primary driver	[117,118,119]
Selenium	Component of selenoproteins (GPx, thioredoxin reductase); central in redox buffering and ROS detoxification	Mixed evidence; RCT showed ↑ risk of T2D with long-term supplementation; non-linear effects	Low consistency/controversial	Illustrates redox hormesis vs. excess paradox; both deficiency and excess impair metabolic regulation; highlights need for homeostatic balance	[120,121,122]
Chromium	Proposed enhancer of insulin signaling; may influence glucose uptake and disposal	Inconsistent results across RCTs; modest or null effects on glycemia and lipids	Low consistency	Represents gap between mechanistic plausibility vs. clinical efficacy; not robust as intervention; relevance mainly theoretical	[123,124]
Copper	Cofactor in antioxidant enzymes (Cu/Zn-SOD); involved in redox reactions and oxidative balance	Limited direct metabolic RCT evidence; mechanistic support strong	Low–moderate (mechanistic)	Dual role: essential for antioxidant defense but pro-oxidant if imbalanced; matrix-dependent relevance	[125]
Iron	Oxygen transport; mitochondrial respiration; redox-active metal (Fenton chemistry)	Excess iron linked to insulin resistance, inflammation, hepatic dysfunction	Moderate consistency (observational + mechanistic)	Key example of redox overload: excess amplifies oxidative stress and metabolic dysfunction; not universally beneficial	[54]

## Data Availability

The original contributions presented in this study are included in the article. Further inquiries can be directed to the corresponding author.

## References

[1] Robins D.J. (1987). Pyrrolizidine alkaloids. Nat. Prod. Rep..

[2] Moreira R., Pereira D.M., Valentao P., Andrade P.B. (2018). Pyrrolizidine Alkaloids: Chemistry, Pharmacology, Toxicology and Food Safety. Int. J. Mol. Sci..

[3] Robertson J., Stevens K. (2017). Pyrrolizidine alkaloids: Occurrence, biology, and chemical synthesis. Nat. Prod. Rep..

[4] Xiong F., Jiang K.Y., Chen Y., Ju Z.C., Yang L., Xiong A.Z., Wang Z.T. (2020). Protein cross-linking in primary cultured mouse hepatocytes by dehydropyrrolizidine alkaloids: Structure-toxicity relationship. Toxicon.

[5] Yang M.B., Ruan J.Q., Gao H., Li N., Ma J., Xue J.Y., Ye Y., Fu P.P.C., Wang J.Y., Lin G. (2017). First evidence of pyrrolizidine alkaloid N-oxide-induced hepatic sinusoidal obstruction syndrome in humans. Arch. Toxicol..

[6] Teschke R., Vongdala N., Quan N.V., Quy T.N., Xuan T.D. (2021). Metabolic Toxification of 1,2-Unsaturated Pyrrolizidine Alkaloids Causes Human Hepatic Sinusoidal Obstruction Syndrome: The Update. Int. J. Mol. Sci..

[7] Jiao W.-T., Zhu L., Shen T.-T., Wang L.-Y., Li Q.X., Wang C., Wu X.-W., Chen H.-P., Hua R.-M. (2024). Simultaneous determination of 15 pyrrolizidine alkaloids and their N-oxides in weeds, soil, fresh tea leaves, and tea: Exploring the pollution source of pyrrolizidine alkaloids in tea. Food Chem..

[8] Nowak M., Wittke C., Lederer I., Klier B., Kleinwächter M., Selmar D. (2016). Interspecific transfer of pyrrolizidine alkaloids: An unconsidered source of contaminations of phytopharmaceuticals and plant derived commodities. Food Chem..

[9] Selmar D., Wittke C., Beck-von Wolffersdorff I., Klier B., Lewerenz L., Kleinwächter M., Nowak M. (2019). Transfer of pyrrolizidine alkaloids between living plants: A disregarded source of contaminations. Environ. Pollut..

[10] Zhu L., Xue J.Y., Xia Q.S., Fu P.P., Lin G. (2017). The long persistence of pyrrolizidine alkaloid-derived DNA adducts in vivo: Kinetic study following single and multiple exposures in male ICR mice. Arch. Toxicol..

[11] Song Z.J., He Y.S., Ma J., Fu P.P., Lin G. (2020). Pulmonary toxicity is a common phenomenon of toxic pyrrolizidine alkaloids. J. Environ. Sci. Health Part C—Toxicol. Carcinog..

[12] He Y.S., Zhu L., Ma J., Lin G. (2021). Metabolism-mediated cytotoxicity and genotoxicity of pyrrolizidine alkaloids. Arch. Toxicol..

[13] Schoental R. (1961). Liver Changes and Primary Liver Tumours in Rats Given Toxic Guinea Pig Diet (M.R.C. Diet 18). Br. J. Cancer.

[14] Yang M.B., Ruan J.Q., Fu P.P., Lin G. (2016). Cytotoxicity of pyrrolizidine alkaloid in human hepatic parenchymal and sinusoidal endothelial cells: Firm evidence for the reactive metabolites mediated pyrrolizidine alkaloid-induced hepatotoxicity. Chem.-Biol. Interact..

[15] Ruan J.Q., Liao C.S., Ye Y., Lin G. (2014). Lack of Metabolic Activation and Predominant Formation of an Excreted Metabolite of Nontoxic Platynecine-Type Pyrrolizidine Alkaloids. Chem. Res. Toxicol..

[16] Li N., Xia Q.S., Ruan J.Q., Fu P.P., Lin G. (2011). Hepatotoxicity and Tumorigenicity Induced by Metabolic Activation of Pyrrolizidine Alkaloids in Herbs. Curr. Drug Metab..

[17] Lin G., Wang J.Y., Li N., Li M., Gao H., Ji Y.A., Zhang F., Wang H.L., Zhou Y., Ye Y. (2011). Hepatic sinusoidal obstruction syndrome associated with consumption of *Gynura segetum*. J. Hepatol..

[18] Zhuge Y.Z., Liu Y.L., Xie W.F., Zou X.P., Xu J.M., Wang J.Y., Bai W.Y., Chen D.F., Chen J., Chen L.G. (2019). Expert consensus on the clinical management of pyrrolizidine alkaloid-induced hepatic sinusoidal obstruction syndrome. J. Gastroenterol. Hepatol..

[19] Rech C., Ribeiro L.D., Bento J.M.S., Crevelin E.J., Pott C.A., Nardi C. (2025). *Crotalaria juncea* reduces larval survival and adult fecundity of *Diabrotica speciosa*. J. Pest Sci..

[20] Eröksüz Y., Çeribasi A.O., Çevik A., Eröksüz H., Tosun F., Tamer U. (2008). Toxicity of *Heliotropium dolosum*, *Heliotropium circinatum*, and *Senecio vernalis* in Parental Quail and Their Progeny, with Residue Evaluation of Eggs. Turk. J. Vet. Anim. Sci..

[21] Cheeke P.R., Pierson-Goeger M.L. (1983). Toxicity of Senecio jacobaea and pyrrolizidine alkaloids in various laboratory animals and avian species. Toxicol. Lett..

[22] Liu F., Rong X.X., Guo H., Xu D., Liu C., Meng L.L., Yang X.Q., Guo T.T., Kan X.F., Song Y.H. (2020). Clinical characteristics, CT signs, and pathological findings of Pyrrolizidine alkaloids-induced sinusoidal obstructive syndrome: A retrospective study. BMC Gastroenterol..

[23] Zhang W., Liu L., Zhang M., Zhang F., Peng C.Y., Zhang B., Chen J., Li L., He J., Xiao J.Q. (2021). Validation of the Nanjing Criteria for Diagnosing Pyrrolizidine Alkaloids-induced Hepatic Sinusoidal Obstruction Syndrome. J. Clin. Transl. Hepatol..

[24] Liang Q.N., Sheng Y.C., Jiang P., Ji L.L., Xia Y.Y., Min Y., Wang Z.T. (2011). The gender-dependent difference of liver GSH antioxidant system in mice and its influence on isoline-induced liver injury. Toxicology.

[25] Huan J.Y., Miranda C.L., Buhler D.R., Cheeke P.R. (1998). Species differences in the hepatic microsomal enzyme metabolism of the pyrrolizidine alkaloids. Toxicol. Lett..

[26] Shubat P.J., Hubbard A.K., Huxtable R.J. (1989). Dose-response relationship in intoxication by the pyrrolizidine alkaloid monocrotaline. J. Toxicol. Environ. Health.

[27] Copple B.L., Ganey P.E., Roth R.A. (2003). Liver inflammation during monocrotaline hepatotoxicity. Toxicology.

[28] Ovelar M.F., García J.A., Cook D., Gardner D., Stegelmeier B., de Ulzurrun P.D., Tettamanti A., Balbuena D., Lita E.V., Poo J.I. (2025). *Senecio pampeanus* poisoning in beef cattle: Case report and toxicological evaluation. Vet. Res. Commun..

[29] Ribeiro M., Bianchi I.N., Silva W.D.M., Cavasani J.P.S., Santos I.G., Dias L., Colodel E.M., Furlan F.H. (2025). Subacute and chronic toxic hepatopathy in cattle grazing pasture with *Crotalaria spectabilis*. Vet. Pathol..

[30] He Y.S., Zhang W., Ma J., Xia Q.S., Song Z.J., Zhu L., Zhang C.Y., Liu J., Ye Y., Fu P.P. (2021). Blood Pyrrole-DNA Adducts Define the Early Tumorigenic Risk in Patients with Pyrrolizidine Alkaloid-Induced Liver Injury. Environ. Sci. Technol. Lett..

[31] Wen C.L., Zhou T., Chang Y.Q., Wei Y., Zhang H.D., Yang Z.F. (2024). Exposure to *Gynura japonica* (Thunb.) Juel plants induces hepatoxicity in rats and Buffalo rat liver cells. J. Ethnopharmacol..

[32] Chen Y., Xiong F., Wang W.Q., Jiang K.Y., Ye X.L., Deng G., Wang C.H., Yang L., Xiong A.Z., Wang Z.T. (2020). The long persistence of pyrrolizidine alkaloid-derived pyrrole-protein adducts in vivo: Kinetic study following multiple exposures of a pyrrolizidine alkaloid containing extract of *Gynura japonica*. Toxicol. Lett..

[33] Xiong A.Z., Shao Y.L., Fang L.X., Yang X., Zhang S.C., Zheng J., Ding W.X., Yang L., Wang Z.T. (2019). Comparative analysis of toxic components in different medicinal parts of *Gynura japonica* and its toxicity assessment on mice. Phytomedicine.

[34] Knoop K., Frahm J., Kersten S., Kluess J., Meyer U., von Soosten D., Beineke A., Saltzmann J., Dänicke S. (2024). Short-term exposure of dairy cows to pyrrolizidine alkaloids from tansy ragwort (*Jacobaea vulgaris* Gaertn.): Effects on organs and indicators of energy metabolism. Arch. Anim. Nutr..

[35] Knoop K., Knappstein K., Kaltner F., Gabler A.M., Taenzer J., These A., Kersten S., Meyer U., Frahm J., Kluess J. (2023). Short-term exposure of dairy cows to pyrrolizidine alkaloids from tansy ragwort (*Jacobaea vulgaris* Gaertn.): Effects on health and performance. Arch. Anim. Nutr..

[36] Zheng Q., Zhang H.Y. (2024). *Gynura segetum* induces hepatic sinusoidal obstruction syndrome in a child: A case report. Medicine.

[37] Barcelos S.T.A., Dall’Oglio V.M., de Araújo A., Cerski C.T.S., Alvares-da-Silva M.R. (2021). Sinusoidal obstruction syndrome secondary the intake of *Senecio brasiliensis*: A case report. Ann. Hepatol..

[38] Panziera W., Gonçalves M.A., Oliveira L.G.S., Lorenzett M.P., Reis M., Harnmerschmitt M.E., Pavarini S.P., Driemeier D. (2017). *Senecio brasiliensis* poisoning in calves: Pattern and evolution of hepatic lesions. Pesqui. Vet. Bras..

[39] Dau S.L., Machado T.P., dos Santos E.D., Setim D.H., Sakis E.R., Alves L.P., da Motta A.C. (2019). Poisoning by *Senecio brasiliensis* in Horses in Northern Rio Grande do Sul. Acta Sci. Vet..

[40] Preliasco M., Gardner D., Moraes J., González A.C., Uriarte G., Rivero R. (2017). *Senecio grisebachii* Baker: Pyrrolizidine alkaloids and experimental poisoning in calves. Toxicon.

[41] Zhang H., Jia S., Jin L.Y., Yao J.Z., Shen Z.H., Wu J.Y., Yao X.K., Chen D.W., Zhang C.C., Yu S.F. (2021). *Gynura segetum* induces hepatic sinusoidal obstruction syndrome in mice by impairing autophagy. Acta Cir. Bras..

[42] Jiang M.J., Wang L.Y., Du X.D., Hao M.M., Gao P.J. (2020). Low molecular weight heparin in the treatment of pyrrolizidine alkaloid-induced hepatic sinusoidal obstruction syndrome: Five case reports. J. Int. Med. Res..

[43] Gu X.Y., Li S.W., Lu M.N., Li Y., Wang Q.X., Chen L., Jia Y.Q., Cao S., Zhang T., Zhou M.M. (2022). Investigation of *Gynura segetum* root extract (GSrE) induced hepatotoxicity based on metabolomic signatures and microbial community profiling in rats. Front. Microbiol..

[44] Zan K., Lei W., Li Y.L., Wang Y., Liu L.A., Zuo T.T., Jin H.Y., Ma S.C. (2022). Integrative Metabolomics and Proteomics Detected Hepatotoxicity in Mice Associated with Alkaloids from *Eupatorium fortunei* Turcz. Toxins.

[45] García J.A., Rosas J.E., Santos C.G.Y., Streitenberger N., Feijoo M., Dutra F. (2020). *Senecio* spp. transboundary introduction and expansion affecting cattle in Uruguay: Clinico-pathological, epidemiological and genetic survey, and experimental intoxication with *Senecio oxyphyllus*. Toxicon.

[46] Canugovi C., Stevenson M.D., Vendrov A.E., Hayami T., Robidoux J., Xiao H., Zhang Y.Y., Eitzman D.T., Runge M.S., Madamanchi N.R. (2019). Increased mitochondrial NADPH oxidase 4 (NOX4) expression in aging is a causative factor in aortic stiffening. Redox Biol..

[47] Panziera W., Bianchi R.M., Mazaro R.D., Giaretta P.R., Silva G.B., Silva D.R.P., Fighera R.A. (2017). Natural poisoning by *Senecio brasiliensis* in horses. Pesqui. Vet. Bras..

[48] Wu D., Gao J. (2018). Hepatic sinusoidal obstruction syndrome related to Tusanqi: Clinical analysis of 19 cases. Advers. Drug React. J..

[49] Leal P.V., de Melo G.K.A., Pott A., Martins T.B., Gardner D., de Barros C.S.L., de Lemos R.A.A. (2019). Hepatic Encephalopathy Secondary to Chronic Liver Lesions Caused by *Crotalaria incana* in a Bovine. Acta Sci. Vet..

[50] Cagin Y.F., Seckin Y., Firat F., Samdanci E. (2017). Acute toxic hepatitis induced by a herbal medicine: Anchusa Boraginaceae. Acta Gastro-Enterol. Belg..

[51] Ji L.L., Zhang M., Sheng Y.C., Wang Z.T. (2005). Pyrrolizidine alkaloid clivorine induces apoptosis in human normal liver L-02 cells and reduces the expression of p53 protein. Toxicol. Vitr..

[52] Haas M., Ackermann G., Küpper J.H., Glatt H., Schrenk D., Fahrer J. (2023). OCT1-dependent uptake of structurally diverse pyrrolizidine alkaloids in human liver cells is crucial for their genotoxic and cytotoxic effects. Arch. Toxicol..

[53] Ruan J.Q., Gao H., Li N., Xue J.Y., Chen J., Ke C.Q., Ye Y., Fu P.P.C., Zheng J., Wang J.Y. (2015). Blood Pyrrole-Protein Adducts-A Biomarker of Pyrrolizidine Alkaloid-Induced Liver Injury in Humans. J. Environ. Sci. Health Part C—Environ. Carcinog. Ecotoxicol. Rev..

[54] Gordon G.J., Coleman W.B., Grisham J.W. (2000). Bax-mediated apoptosis in the livers of rats after partial hepatectomy in the retrorsine model of hepatocellular injury. Hepatology.

[55] Soylu S., Çevik A., Timurkaan N. (2022). Pathological and immunohistochemical changes in the liver of monocrotaline-treated rats. Turk. J. Vet. Anim. Sci..

[56] Ji L.L., Chen Y., Liu T.Y., Wang Z.T.Z. (2008). Involvement of Bcl-xL degradation and mitochondrial-mediated apoptotic pathway in pyrrolizidine alkaloids-induced apoptosis in hepatocytes. Toxicol. Appl. Pharmacol..

[57] Tepe J.J., Williams R.M. (1999). Reductive activation of a hydroxylamine hemiacetal derivative of dehydromonocrotaline: The first reductively activated pyrrolizidine alkaloid capable of cross-linking DNA. Angew. Chem.-Int. Ed..

[58] Hessel-Pras S., Braeuning A., Guenther G., Adawy A., Enge A.M., Ebmeyer J., Henderson C.J., Hengstler J.G., Lampen A., Reif R. (2020). The pyrrolizidine alkaloid senecionine induces CYP-dependent destruction of sinusoidal endothelial cells and cholestasis in mice. Arch. Toxicol..

[59] Yao J., Li C.G., Gong L.K., Feng C.C., Li C.Z., Gao M., Luan Y., Qi X.M., Ren J. (2014). *Hepatic cytochrome* P450s play a major role in monocrotaline-induced renal toxicity in mice. Acta Pharmacol. Sin..

[60] Ji L.L., Zhao X.G., Chen L., Zhang M., Wang Z.T. (2002). Pyrrolizidine alkaloid clivorine inhibits human normal liver L-02 cells growth and activates p38 mitogen-activated protein kinase in L-02 cells. Toxicon.

[61] Rakba N., Melhaoui A., Rissel M., Morel I., Loyer P., Lescoat G. (2000). Irniine, a pyrrolidine alkaloid, isolated from *Arisarum vulgare* can induce apoptosis and/or necrosis in rat hepatocyte cultures. Toxicon.

[62] He Y.Q., Yang L., Liu H.X., Zhang J.W., Liu Y., Fong A., Xiong A.Z., Lu Y.L., Yang L., Wang C.H. (2010). Glucuronidation, a New Metabolic Pathway for Pyrrolizidine Alkaloids. Chem. Res. Toxicol..

[63] Zhu L., Zhang C.Y., Zhang W., Xia Q.S., Ma J., He X., He Y.S., Fu P.P., Jia W., Zhuge Y.Z. (2021). Developing urinary pyrrole-amino acid adducts as non-invasive biomarkers for identifying pyrrolizidine alkaloids-induced liver injury in human. Arch. Toxicol..

[64] He X.B., Xia Q.S., Ma L., Fu P.P. (2016). 7-cysteine-pyrrole conjugate: A new potential DNA reactive metabolite of pyrrolizidine alkaloids. J. Environ. Sci. Health Part C—Environ. Carcinog. Ecotoxicol. Rev..

[65] He X.B., Xia Q.S., Shi Q., Fu P.P. (2020). Effects of glutathione and cysteine on pyrrolizidine alkaloid-induced hepatotoxicity and DNA adduct formation in rat primary hepatocytes. J. Environ. Sci. Health Part C—Toxicol. Carcinog..

[66] He X.B., Xia Q.S., Fu P.P. (2017). 7-Glutathione-pyrrole and 7-cysteine-pyrrole are potential carcinogenic metabolites of pyrrolizidine alkaloids. J. Environ. Sci. Health Part C—Environ. Carcinog. Ecotoxicol. Rev..

[67] Hincks J.R., Kim H.Y., Segall H.J., Molyneux R.J., Stermitz F.R., Coulombe R.A. (1991). DNA cross-linking in mammalian cells by pyrrolizidine alkaloids: Structure-activity relationships. Toxicol. Appl. Pharmacol..

[68] Petry T.W., Bowden G.T., Huxtable R.J., Sipes I.G. (1984). Characterization of hepatic DNA damage induced in rats by the pyrrolizidine alkaloid monocrotaline. Cancer Res..

[69] Petry T.W., Bowden G.T., Buhler D.R., Sipes K.G. (1986). Genotoxicity of the pyrrolizidine alkaloid jacobine in rats. Toxicol. Lett..

[70] Kim H.Y., Stermitz F.R., Li J.K.K., Coulombe R.A. (1999). Comparative DNA cross-linking by activated pyrrolizidine alkaloids. Food Chem. Toxicol..

[71] Abdelfatah S., Nass J., Knorz C., Klauck S.M., Küpper J.H., Efferth T. (2022). Pyrrolizidine alkaloids cause cell cycle and DNA damage repair defects as analyzed by transcriptomics in cytochrome P450 3A4-overexpressing HepG2 clone 9 cells. Cell Biol. Toxicol..

[72] Rieben W.K., Coulombe R.A. (2004). DNA cross-linking by dehydromonocrotaline lacks apparent base sequence preference. Toxicol. Sci..

[73] Allameh A., Niayesh-Mehr R., Aliarab A., Sebastiani G., Pantopoulos K. (2023). Oxidative Stress in Liver Pathophysiology and Disease. Antioxidants.

[74] Wang Z.Q., Han H.L., Wang C., Zheng Q.Q., Chen H.P., Zhang X.C., Hou R.Y. (2021). Hepatotoxicity of Pyrrolizidine Alkaloid Compound Intermedine: Comparison with Other Pyrrolizidine Alkaloids and Its Toxicological Mechanism. Toxins.

[75] Zhu Y.L., Zhang S.H., Shao Y., Tang L.H., Zhang C.C., Tang S.Y., Lu H. (2024). Regulatory role of oxidative stress in retrorsine—Induced apoptosis and autophagy in primary rat hepatocytes. Ecotox. Environ. Safe..

[76] Su L.J., Zhang J.H., Gomez H., Murugan R., Hong X., Xu D.X., Jiang F., Peng Z.Y. (2019). Reactive Oxygen Species-Induced Lipid Peroxidation in Apoptosis, Autophagy, and Ferroptosis. Oxidative Med. Cell. Longev..

[77] Danan G., Teschke R. (2016). RUCAM in Drug and Herb Induced Liver Injury: The Update. Int. J. Mol. Sci..

[78] Zhou Z., Yang D.F., Chu T., Zheng D.Y., Zhang K.Y., Liang S.K., Yang L., Yang Y.C., Ma W.Z. (2026). Hepatic Sinusoidal Obstruction Syndrome Induced by Pyrrolizidine Alkaloids from *Gynura segetum*: Mechanisms and Therapeutic Advances. Molecules.

[79] Lin G., Cui Y.Y., Liu X.Q., Wang Z.T. (2002). Species differences in the in vitro metabolic activation of the hepatotoxic pyrrolizidine alkaloid clivorine. Chem. Res. Toxicol..

[80] Lakshmanan H., Raman J., Pandian A., Kuppamuthu K., Nanjian R., Sabaratam V., Naidu M. (2016). Aqueous extract of Senecio candicans DC induce liver and kidney damage in a sub-chronic oral toxicity study in Wistar rats. Regul. Toxicol. Pharmacol..

[81] Dueker S.R., Lame M.W., Segall H.J. (1992). Hydrolysis of pyrrolizidine alkaloids by guinea pig hepatic carboxylesterases. Toxicol. Appl. Pharmacol..

[82] Mattocks A.R. (1982). Hydrolysis and hepatotoxicity of retronecine diesters. Toxicol. Lett..

[83] Chung W.G., Buhler D.R. (2004). Differential metabolism of the pyrrolizidine alkaloid, senecionine, in Fischer 344 and Sprague-Dawley rats. Arch. Pharm. Res..

[84] Chung W.G., Buhler D.R. (1995). Major factors for the susceptibility of guinea pig to the pyrrolizidine alkaloid jacobine. Drug Metab. Dispos..

[85] Duringer J.M., Buhler D.R., Craig A.M. (2004). Comparison of hepatic in vitro metabolism of the pyrrolizidine alkaloid senecionine in sheep and cattle. Am. J. Vet. Res..

[86] Yang M.B., Ma J., Ruan J.Q., Ye Y., Fu P.P.C., Lin G. (2019). Intestinal and hepatic biotransformation of pyrrolizidine alkaloid *N*-oxides to toxic pyrrolizidine alkaloids. Arch. Toxicol..

[87] Fu P.P., Xia Q.S., Lin G., Chou M.W. (2002). Genotoxic Pyrrolizidine Alkaloids—Mechanisms Leading to DNA Adduct Formation and Tumorigenicity. Int. J. Mol. Sci..

[88] Fu P.P., Xia Q.S., Lin G., Chou M.W. (2004). Pyrrolizidine alkaloids—Genotoxicity, metabolism enzymes, metabolic activation, and mechanisms. Drug Metab. Rev..

[89] Wang W.Q., Chen Y., Yin Y., Wang X.J., Ye X.L., Jiang K.Y., Zhang Y., Zhang J.W., Zhang W., Zhuge Y.Z. (2022). A TMT-based shotgun proteomics uncovers overexpression of thrombospondin 1 as a contributor in pyrrolizidine alkaloid-induced hepatic sinusoidal obstruction syndrome. Arch. Toxicol..

[90] Li Y.H., Tai W.C.S., Xue J.Y., Wong W.Y., Lu C., Ruan J.Q., Li N., Wan T.F., Chan W.Y., Hsiao W.L.W. (2015). Proteomic Study of Pyrrolizidine Alkaloid-Induced Hepatic Sinusoidal Obstruction Syndrome in Rats. Chem. Res. Toxicol..

[91] Chen Y., Wang W.Q., Jia X.L., Wang C.H., Yang L., Wang Z.T., Xiong A.Z. (2022). Firm evidence for the detoxification of senecionine-induced hepatotoxicity via N-glucuronidation in UGT1A4-humanized transgenic mice. Food Chem. Toxicol..

[92] Tang J., Akao T., Nakamura N., Wang Z.T., Takagawa K., Sasahara M., Hattori M. (2007). In vitro metabolism of isoline, a pyrrolizidine alkaloid from *Ligularia duciformis*, by rodent liver microsomal esterase and enhanced hepatotoxicity by esterase inhibitors. Drug Metab. Dispos..

[93] Geburek I., Schrenk D., These A. (2020). In vitro biotransformation of pyrrolizidine alkaloids in different species: Part II-identification and quantitative assessment of the metabolite profile of six structurally different pyrrolizidine alkaloids. Arch. Toxicol..

[94] Edgar J.A., Molyneux R.J., Colegate S.M. (2015). Pyrrolizidine Alkaloids: Potential Role in the Etiology of Cancers, Pulmonary Hypertension, Congenital Anomalies, and Liver Disease. Chem. Res. Toxicol..

[95] Xia Q.S., Zhao Y.W., Von Tungeln L.S., Doerge D.R., Lin G., Cai L.N., Fu P.P. (2013). Pyrrolizidine Alkaloid-Derived DNA Adducts as a Common Biological Biomarker of Pyrrolizidine Alkaloid-Induced Tumorigenicity. Chem. Res. Toxicol..

[96] Zhao Y.W., Xia Q.S., da Costa G.G., Yu H.T., Cai L.N., Fu P.P. (2012). Full Structure Assignments of Pyrrolizidine Alkaloid DNA Adducts and Mechanism of Tumor Initiation. Chem. Res. Toxicol..

[97] Lu Y., Ma J., Song Z.J., Ye Y., Fu P.P., Lin G. (2018). The role of formation of pyrrole-ATP synthase subunit beta adduct in pyrrolizidine alkaloid-induced hepatotoxicity. Arch. Toxicol..

[98] Ma J., Xia Q.S., Fu P.P., Lin G. (2018). Pyrrole-protein adducts—A biomarker of pyrrolizidine alkaloid-induced hepatotoxicity. J. Food Drug Anal..

[99] Gao H., Ruan J.Q.Q., Chen J., Li N., Ke C.Q.Q., Ye Y., Lin G., Wang J.Y.Y. (2015). Blood pyrrole-protein adducts as a diagnostic and prognostic index in pyrrolizidine alkaloid-hepatic sinusoidal obstruction syndrome. Drug Des. Dev. Ther..

[100] Xia Q.S., Zhao Y.W., Lin G., Beland F.A., Cai L.N., Fu P.P. (2016). Pyrrolizidine Alkaloid-Protein Adducts: Potential Non-invasive Biomarkers of Pyrrolizidine Alkaloid-Induced Liver Toxicity and Exposure. Chem. Res. Toxicol..

[101] Li W.W., Cheng T., Jiang T.T., Zhou M.Y., Gong B.W., Zhao G.D., Li J., Tan R., Yang X.J., Joshi K. (2022). Hepatic RNA adduction derived from metabolic activation of retrorsine in vitro and in vivo. Chem.-Biol. Interact..

[102] Chen M.X., Li L., Zhong D.F., Shen S.J., Zheng J., Chen X.Y. (2016). 9-Glutathiony1-6,7-dihydro-1-hydroxymethy1-5H-pyrrolizine Is the Major Pyrrolic Glutathione Conjugate of Retronecine-Type Pyrrolizidine Alkaloids in Liver Microsomes and in Rats. Chem. Res. Toxicol..

[103] Kim H.Y., Stermitz F.R., Coulombe R.A. (1995). Pyrrolizidine alkaloid-induced DNA-protein cross-links. Carcinogenesis.

[104] Thomas H.C., Lame M.W., Wilson D.W., Segall H.J. (1996). Cell cycle alterations associated with covalent binding of monocrotaline pyrrole to pulmonary artery endothelial cell DNA. Toxicol. Appl. Pharmacol..

[105] Tang Y.H., Li J., Gao C., Xu Y.Y., Li Y.Y., Yu X., Wang J., Liu L.G., Yao P. (2016). Hepatoprotective Effect of Quercetin on Endoplasmic Reticulum Stress and Inflammation after Intense Exercise in Mice through Phosphoinositide 3-Kinase and Nuclear Factor-Kappa B. Oxidative Med. Cell. Longev..

[106] Ji L.L., Liu T.Y., Wang Z.T. (2010). Pyrrolizidine alkaloid clivorine induced oxidative injury on primary cultured rat hepatocytes. Hum. Exp. Toxicol..

[107] Liu T.Y., Chen Y., Wang Z.Y., Ji L.L., Wang Z.T. (2010). Pyrrolizidine alkaloid isoline-induced oxidative injury in various mouse tissues. Exp. Toxicol. Pathol..

[108] Wang Z.Y., Kang H., Ji L.L., Yang Y.Q., Liu T.Y., Cao Z.W., Morahan G., Wang Z.T. (2012). Proteomic characterization of the possible molecular targets of pyrrolizidine alkaloid isoline-induced hepatotoxicity. Environ. Toxicol. Pharmacol..

[109] Yan X.M., Kang H., Feng J., Yang Y.Y., Tang K.L., Zhu R.X., Yang L., Wang Z.T., Cao Z.W. (2016). Identification of Toxic Pyrrolizidine Alkaloids and Their Common Hepatotoxicity Mechanism. Int. J. Mol. Sci..

[110] Li D.P., Chen Y.L., Jiang H.Y., Chen Y., Zeng X.Q., Xu L.L., Ye Y., Ke C.Q., Lin G., Wang J.Y. (2019). Phosphocreatine attenuates *Gynura segetum*-induced hepatocyte apoptosis via a SIRT3-SOD2-mitochondrial reactive oxygen species pathway. Drug Des. Dev. Ther..

[111] Bause A.S., Haigis M.C. (2013). SIRT3 regulation of mitochondrial oxidative stress. Exp. Gerontol..

[112] Tonelli C., Chio I.I.C., Tuveson D.A. (2018). Transcriptional Regulation by Nrf2. Antioxid. Redox Signal..

[113] Huang Z.L., Chen M.W., Wei M.J., Lu B., Wu X.J., Wang Z.T., Ji L.L. (2019). Liver Inflammatory Injury Initiated by DAMPs-TLR4-MyD88/TRIF-NFκB Signaling Pathway Is Involved in Monocrotaline-Induced HSOS. Toxicol. Sci..

[114] Zhang J.Q., Sheng Y.C., Shi L., Zheng Z.Y., Chen M.W., Lu B., Ji L.J. (2017). Quercetin and baicalein suppress monocrotaline-induced hepatic sinusoidal obstruction syndrome in rats. Eur. J. Pharmacol..

[115] Wang Z.T., Ma J., He Y.S., Miu K.K., Yao S., Tang C.P., Ye Y., Lin G. (2022). Nrf2-mediated liver protection by 18β-glycyrrhetinic acid against pyrrolizidine alkaloid-induced toxicity through PI3K/Akt/GSK3β pathway. Phytomedicine.

[116] Shang H.T., Huang C., Xiao Z.L., Yang P.C., Zhang S.Y., Hou X.H., Zhang L. (2023). Gut microbiota-derived tryptophan metabolites alleviate liver injury via AhR/Nrf2 activation in pyrrolizidine alkaloids-induced sinusoidal obstruction syndrome. Cell Biosci..

[117] Dukhande V.V., Malthankar-Phatak G.H., Hugus J.J., Daniels C.K., Lai J.C.K. (2006). Manganese-induced neurotoxicity is differentially enhanced by glutathione depletion in astrocytoma and neuroblastoma cells. Neurochem. Res..

[118] Ji L.L., Chen Y., Wang Z.T. (2008). Intracellular glutathione plays important roles in pyrrolizidine alkaloid clivorine-induced toxicity on L-02 hepatocytes. Toxicol. Mech. Methods.

[119] Chen Y., Ji L.L., Wang H.T., Wang Z.T. (2009). Intracellular glutathione plays important roles in pyrrolizidine alkaloids-induced growth inhibition on hepatocytes. Environ. Toxicol. Pharmacol..

[120] Ji L.L., Chen Y., Wang Z.T. (2008). Protection of *S*-adenosyl methionine against the toxicity of clivorine on hepatocytes. Environ. Toxicol. Pharmacol..

[121] Steenkamp V., Stewart M.J., van der Merwe S., Zuckerman M., Crowther N.J. (2001). The effect of *Senecio latifolius* a plant used as a South African traditional medicine, on a human hepatoma cell line. J. Ethnopharmacol..

[122] Ji L.L., Liu T.Y., Chen Y., Wang Z.T. (2009). Protective Mechanisms of *N*-Acetyl-Cysteine Against Pyrrolizidine Alkaloid Clivorine-Induced Hepatotoxicity. J. Cell. Biochem..

[123] Yee S.B., Kinser S., Hill D.A., Barton C.C., Hotchkiss J.A., Harkema J.R., Ganey P.E., Roth R.A. (2000). Synergistic hepatotoxicity from coexposure to bacterial endotoxin and the pyrrolizidine alkaloid monocrotaline. Toxicol. Appl. Pharmacol..

[124] Copple B.L., Rondelli C.M., Maddox J.F., Hoglen N.C., Ganey P.E., Roth R.A. (2004). Modes of cell death in rat liver after monocrotaline exposure. Toxicol. Sci..

[125] Lossi L. (2022). The concept of intrinsic versus extrinsic apoptosis. Biochem. J..

[126] Ebmeyer J., Rasinger J.D., Hengstler J.G., Schaudien D., Creutzenberg O., Lampen A., Braeuning A., Hessel-Pras S. (2020). Hepatotoxic pyrrolizidine alkaloids induce DNA damage response in rat liver in a 28-day feeding study. Arch. Toxicol..

[127] Gao X.G., Wei M.X., Shan W.H., Liu Q., Gao J., Liu Y., Zhu S.L., Yao H. (2019). An oral 2-hydroxypropyl-β-cyclodextrin-loaded spirooxindole-pyrrolizidine derivative restores p53 activity via targeting MDM2 and JNK1/2 in hepatocellular carcinoma. Pharmacol. Res..

[128] Galluzzi L., Vitale I., Aaronson S.A., Abrams J.M., Adam D., Agostinis P., Alnemri E.S., Altucci L., Amelio I., Andrews D.W. (2018). Molecular mechanisms of cell death: Recommendations of the Nomenclature Committee on Cell Death 2018. Cell Death Differ..

[129] Waizenegger J., Braeuning A., Templin M., Lampen A., Hessel-Pras S. (2018). Structure-dependent induction of apoptosis by hepatotoxic pyrrolizidine alkaloids in the human hepatoma cell line HepaRG: Single versus repeated exposure. Food Chem. Toxicol..

[130] Cassidy-Stone A., Chipuk J.E., Ingerman E., Song C., Yoo C., Kuwana T., Kurth M.J., Shaw J.T., Hinshaw J.E., Green D.R. (2008). Chemical inhibition of the mitochondrial division dynamin reveals its role in Bax/Bak-dependent mitochondrial outer membrane permeabilization. Dev. Cell.

[131] Wang W.Q., Yang X., Chen Y., Ye X.L., Jiang K.Y., Xiong A.Z., Yang L., Wang Z.T. (2020). Seneciphylline, a main pyrrolizidine alkaloid in *Gynura japonica*, induces hepatotoxicity in mice and primary hepatocytes via activating mitochondria-mediated apoptosis. J. Appl. Toxicol..

